# AJGM: joint learning of heterogeneous gene networks with adaptive graphical model

**DOI:** 10.1093/bioinformatics/btaf096

**Published:** 2025-03-12

**Authors:** Shunqi Yang, Lingyi Hu, Pengzhou Chen, Xiangxiang Zeng, Shanjun Mao

**Affiliations:** Department of Statistics, Hunan University, Changsha, Hunan, 410006, China; Department of Statistics, Hunan University, Changsha, Hunan, 410006, China; Department of Statistics, Hunan University, Changsha, Hunan, 410006, China; College of Computer Science and Electronic Engineering, Hunan University, Changsha, Hunan, 410082, China; Department of Statistics, Hunan University, Changsha, Hunan, 410006, China

## Abstract

**Motivation:**

Inferring gene networks provides insights into biological pathways and functional relationships among genes. When gene expression samples exhibit heterogeneity, they may originate from unknown subtypes, prompting the utilization of mixture Gaussian graphical model (GGM) for simultaneous subclassification and gene network inference. However, this method overlooks the heterogeneity of network relationships across subtypes and does not sufficiently emphasize shared relationships. Additionally, GGM assumes data follows a multivariate Gaussian distribution, which is often not the case with zero-inflated scRNA-seq data.

**Results:**

We propose an Adaptive Joint Graphical Model (AJGM) for estimating multiple gene networks from single-cell or bulk data with unknown heterogeneity. In AJGM, an overall network is introduced to capture relationships shared by all samples. The model establishes connections between the subtype networks and the overall network through adaptive weights, enabling it to focus more effectively on gene relationships shared across all networks, thereby enhancing the accuracy of network estimation. On synthetic data, the proposed approach outperforms existing methods in terms of sample classification and network inference, particularly excelling in the identification of shared relationships. Applying this method to gene expression data from triple-negative breast cancer confirms known gene pathways and hub genes, while also revealing novel biological insights.

**Availability and implementation:**

The Python code and demonstrations of the proposed approaches are available at https://github.com/yyytim/AJGM, and the software is archived in Zenodo with DOI: 10.5281/zenodo.14740972.

## 1 Introduction

Gene networks play a crucial role in understanding functional relationships and biological patterns among genes (Saha *et al.* 2017). Several statistical models have been proposed to infer gene networks from gene expression data, based on methods such as correlation (Tan *et al.* 2022), mutual information (Zhao *et al.* 2016), regression (Duren *et al.* 2017), and probabilistic graphical models (Wu et al., 2017). Among these, Gaussian graphical models (GGMs) are widely favored for their ability to capture conditional dependencies between variables. This feature allows them to more accurately reflect real-world interactions. GGM assumes that gene expression data follow a multivariate Gaussian distribution, and significant conditional dependencies among genes are identified by estimating the inverse of the covariance matrix, also called the precision matrix. The non-zero off-diagonal elements of the precision matrix represent edges in the network.

In multi-condition gene expression studies, co-expression profiles across conditions are often related, making it essential to investigate both similarities and differences in gene networks across conditions (El-Kebir *et al.* 2015, Ficklin *et al.* 2017). Both shared and condition-specific relationships carry significant biological implications for understanding the heterogeneity and commonality within these networks. In this context, one method for identifying shared patterns involves constructing a regularization function that encourages similarities between graphs, a technique known as joint estimation. It has been shown that joint estimation can avoid suboptimal solutions and detect edges that may be missed in independent estimation (Lee and Liu, 2015). Among frequentist methods, the joint graphical lasso (JGL) (Danaher *et al.* 2014) is the most prominent approach for achieving this. JGL utilizes two distinct forms of convex penalty functions: one promotes structural similarity by enforcing similarity in precision matrices, while the other encourages shared sparse structures across subnetworks using a group lasso penalty. In the Bayesian paradigm, inference methods for GGMs that utilize different prior distributions have proven effective for high-dimensional sparse data (Williams 2021).

In certain cases, prior knowledge about the class membership is unavailable, preventing the direct application of the aforementioned methods for network inference. For instance, scRNA-seq technology can detect random gene expression variability within a single population (Papalexi and Satija, 2018), but the precise number and classification of subpopulations often remain undetermined. When heterogeneity is unknown, certain methods integrate graphical models with mixture distributions to accomplish concurrent clustering and network estimation (Gao *et al.* 2016). Compared to methods that first perform sample clustering followed by joint estimation, these approaches allow for mutual adjustment between clustering and network inference (Tan 2022), resulting in more coherent and accurate identification of subtypes and their interactions.

The essence of joint estimation lies in measuring relationships between networks by examining partially shared edge structures. However, existing methods present several limitations in assessing these relationships. First, these methods inherently assume equal similarity among networks across subtypes, an assumption that is problematic due to the heterogeneity in genes and subtypes. To address this issue, the Condition-adaptive Fused Graphical Lasso (CFGL) (Lyu *et al.* 2018) is proposed, which allows for adaptive adjustment of similarity in gene relationships. However, CFGL adjusts similarity weights using binary variables, which fails to capture the precise degree of similarity between networks. Second, shared relationships across subtypes are derived by promoting similarity between precision matrices during joint estimation and then extracting relationships common to all networks. This method derives shared relationships through pairwise network connections but lacks sufficient consideration of the overall sample context.

**Figure 1.**
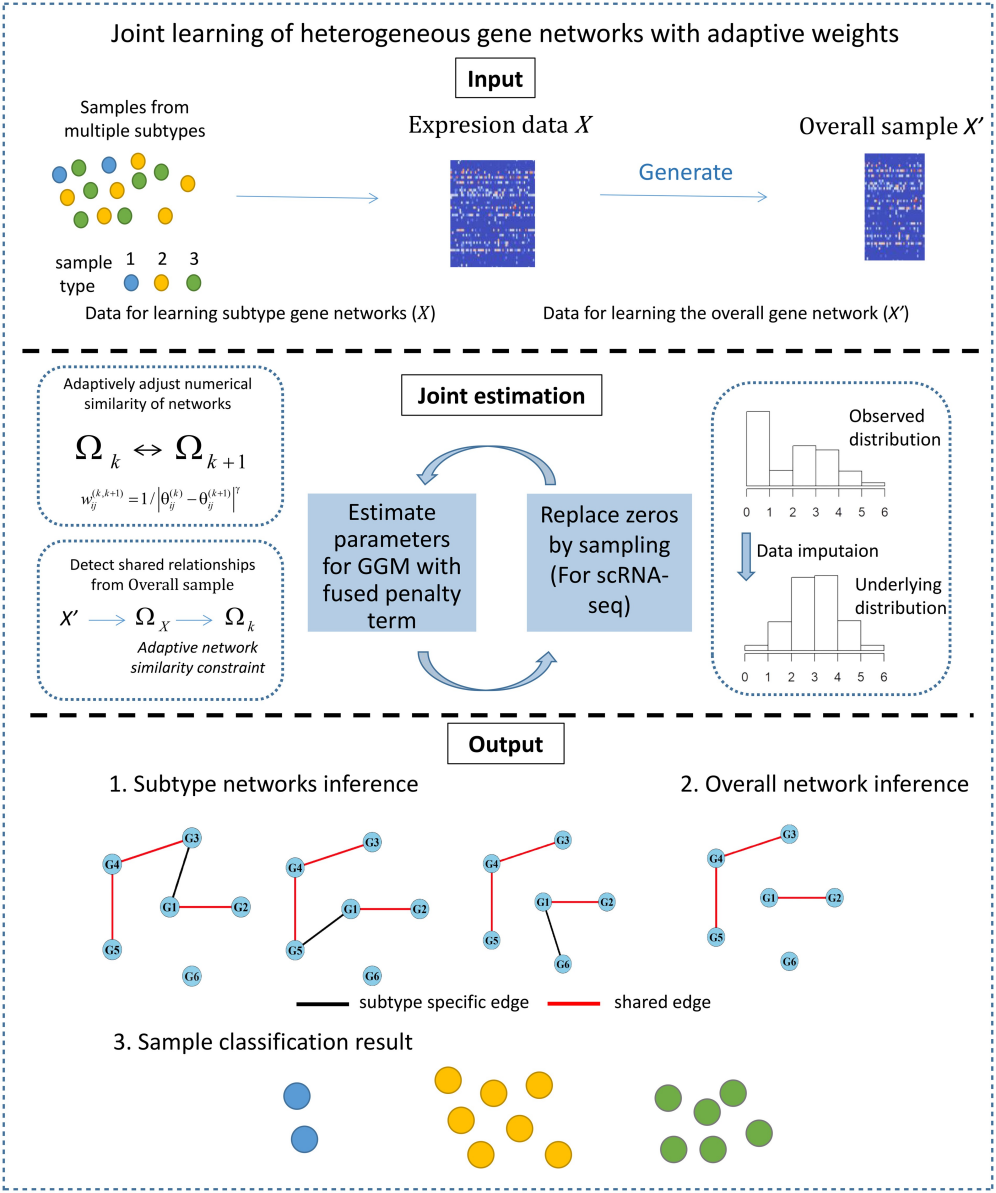
The flowchart of the Adaptive Joint Graphical Model (AJGM). The model inputs include expression data and an overall sample derived from the expression data, which can be generated using various methods. The parameter estimation process involves: (i) dynamically adjusting the similarity relationships between networks during the joint estimation of GGMs, (ii) identifying shared relationships between networks from the overall sample, and (iii) implementing a data imputation process tailored for zero-inflated data. The model outputs include both the clustering results of the samples and the inferred network structures.

Considering the limitations of existing models, we propose a new method named Adaptive Joint Graphical Model (AJGM) for jointly estimating multiple gene networks, with its flowchart shown in Fig. 1. AJGM can simultaneously classify samples and infer networks, while adaptively adjusting the similarity between network structures. By incorporating the overall network in the joint estimation, the model’s ability to identify shared relationships is significantly improved, thus enhancing the estimation accuracy of gene networks. Furthermore, to address the challenge posed by a large number of zeros in single-cell data, we develop a data imputation method that extends the GGM’s applicability to both scRNA-seq data and bulk RNA-seq data. The gene networks estimated by AJGM outperform comparative methods on simulated data and yield biologically meaningful results in gene expression data of triple-negative breast cancer.

## 2 Models

### 2.1 Modeling subtype gene networks

Suppose that there are n samples, each with p genes in expression data X. Considering the heterogeneity, we assume the n samples belong to K subtypes. Therefore, the samples $x_{l}^{\prime },l=1,\ldots ,n$ come from a Gaussian mixture distribution with the probability density function:

(1) $$p(x_{l})=\sum_{k=1}^{K}\pi_{k}N(x_{l}|\mu_{k},\Omega_{k}{}^{-1}),$$

where N denotes the multivariate normal density, l is the sample index, $\pi_{k}$ is the proportion of subtype k with $\sum_{k=1}^{K}\pi_{k}=1$, $\mu_{k}$ denotes the mean, and $\Omega_{k}$ represents the precision matrix of k. The non-zero elements in $\Omega_{k}$ represent the connected edges in the gene network of subtype k.

### 2.2 Modeling the overall gene network

To improve the model’s ability to recognize shared relationships, we introduce an overall gene network in joint learning. The overall network can identify shared relationships and make these edges more likely to appear in all subtype networks during joint estimation.

When modeling the overall gene network, we no longer consider the samples to belong to different subtypes. We set $X^{\prime }$ as overall samples of the original data X for learning the overall gene network and $x_{t}^{\prime },t=1,\ldots ,m$ is assumed to follow a normal distribution:

(2) $$p\left(x_{t}^{\prime }\right)=N\left(x_{t}^{\prime }|\mu_{X},\Omega_{X}{}^{-1}\right).$$

The overall network $\Omega_{X}$ captures shared relationships by using maximum likelihood estimation, which emphasizes patterns and connections that are consistent across the majority of samples. In addition, the overall network serves to identify potential shared relationships and facilitates their presence in subtype-specific networks. The final estimated network of shared relationships is obtained by taking the union of all subtype-specific networks.

There are multiple methods to obtain $X^{\prime }$. When specific overall samples are available, they can be directly utilized. In cases where such information is lacking, we recommend using the following two methods to obtain $X^{\prime }$:

1. **Setting**  $X^{\prime }$  **as**  X. A straightforward idea is to designate all samples in $X^{\prime }$ as X, since this approach can capture the complete information for learning shared relationships. In this case, m, representing the number of overall samples, is equal to n. However, setting $X^{\prime }$ as X does not conform to the definition of the likelihood function in joint estimation as samples cannot simultaneously appear in two terms. Despite this theoretical gap, this method demonstrates relatively high accuracy in simulation study.
2. **Obtaining**  $X^{\prime }$  **by sampling.** We can generate $X^{\prime }$ by sampling from a normal distribution. Assuming that X follows a multivariate normal distribution, we calculate parameters of X (mean and covariance matrix), and sample data from the distribution to generate $X^{\prime }$. Here, m is the number of samples chosen during the sampling process.

### 2.3 The unified model in joint estimation

Given expression data X and overall samples $X^{\prime }$, the parameters to be estimated Θ and the likelihood function are

$$\Theta =\left(\pi_{1},\ldots ,\pi_{K},\mu_{1},\ldots ,\mu_{K},\mu_{X},\Omega_{1},\ldots ,\Omega_{K},\Omega_{X}\right)$$

 

(3) $$\begin{array}{c} \;\text{log}\;L(\Theta )=\sum_{l=1}^{n}\text{log}\left\{\sum_{k=1}^{K}\pi_{k}N(x_{l}|\mu_{k},{\Omega_{k}}^{-1})\right\}\\ +\sum_{t=1}^{m}\;\text{log}\;\left\{N(x_{t}^{\prime }|\mu_{X},\Omega_{X}{}^{-1})\right\}-P(\Theta ) \end{array}.$$

Considering the sparsity and similarity of networks’ topology, we propose the following fused penalty term with adaptive weights,

(4) $$\begin{array}{c} P(\Theta )=\lambda_{1}\left(\sum_{i\neq j}\sum_{k=1}^{K}\left|\theta_{ij}^{\left(k\right)}\right|+\sum_{i\neq j}\left|\theta_{ij}^{\left(X\right)}\right|\right)\\ +\lambda_{2}\sum_{i\neq j}\sum_{k=1}^{K-1}w_{ij}^{\left(k,k+1\right)}\left|\theta_{ij}^{\left(k\right)}-\theta_{ij}^{\left(k+1\right)}\right|\\ +\lambda_{3}\sum_{i\neq j}f(\theta_{ij}^{\left(1\right)},\ldots .,\theta_{ij}^{\left(k\right)})w_{ij}^{\left(K,X\right)}\left|\theta_{ij}^{\left(K\right)}-\theta_{ij}^{\left(X\right)}\right| \end{array},$$

 

(5) $$w_{ij}^{\left(a,b\right)}=1/{\left|\theta_{ij}^{\left(a\right)}-\theta_{ij}^{\left(b\right)}\right|}^{\gamma },$$

 

(6) $$f(\theta_{ij}^{\left(1\right)},\ldots .,\theta_{ij}^{\left(k\right)})=\{\begin{array}{c} 1\left(\left|\theta_{ij}^{\left(k\right)}-\theta_{ij}^{\left(k+1\right)}\right|<\mathrm{tre}\right)\\ \frac{\lambda_{2}}{\lambda_{3}}\;\text{else} \end{array},$$

where $\theta_{ij}^{\left(k\right)}$ and $\theta_{ij}^{\left(X\right)}$ denote the (i,j)-th elements of the precision matrix for subtype *k* and overall samples $x^{\prime }$, respectively. $w_{ij}^{\left(k,k+1\right)}$ and $f(\theta_{ij}^{\left(1\right)},\ldots .,\theta_{ij}^{\left(k\right)})$ are adaptive weights. The entire penalty term can be summarized into the following two parts:

1. **Fused penalty term:** In the penalty term, $\lambda_{1}$ is a non-negative tuning parameter that controls the sparsity of the network (Yuan and Lin 2007). $\lambda_{2}$ is a non-negative tuning parameter that controls the similarity of subtype networks. $\lambda_{3}$ is a tuning parameter greater than $\lambda_{2}$, adjusting the similarity between the overall network and subtypes networks. It penalizes the differences between $\Omega_{K}$ and $\Omega_{X}$. With the inclusion of the second penalty term, the similarity between $\Omega_{K}$ and $\Omega_{X}$ can be chained through the two-by-two similarity between subtypes (Seal *et al.* 2023). This approach ensures that subtype gene networks are more likely to retain gene relationships in overall network, consequently enhancing the accuracy of detecting shared relationships.
2. **Adaptive weights:** The adaptive weights in AJGM consist of two parts. First, $w_{ij}^{\left(a,b\right)}$ are the weights imposed on penalizing the pairwise differences between precision matrices. If $\left|\theta_{ij}^{\left(a\right)}-\theta_{ij}^{\left(b\right)}\right|$ is large, suggesting that the relationship between gene *i* and gene *j* differs significantly between the two networks, we apply a smaller weight to the penalty term. Conversely, if $\left|\theta_{ij}^{\left(a\right)}-\theta_{ij}^{\left(b\right)}\right|$ is small, we apply a larger weight to the penalty term to encourage similarity between networks. γ is a non-negative tuning parameter that adjusts the weight. Second, since the model should place more emphasis on relationships shared by all samples, we introduce $f(\theta_{ij}^{\left(1\right)},\ldots .,\theta ij^{\left(k\right)})$ to regulate the alignment between the overall network and the subtype networks. *tre* represents a non-negative threshold that determines the similarity of values in the precision matrices. When the following condition holds:
  $$\forall k\in \left\{1,2,\ldots .,K-1\right\},\left(\left|\theta_{ij}^{\left(k\right)}-\theta_{ij}^{\left(k+1\right)}\right|<\mathrm{tre}\right)$$
  the differences between precision matrices are minimal. In this case, as the relationship between gene i and gene j is likely to be consistent across subtypes, we apply a larger parameter $\lambda_{3}$ to this penalty term, promoting similarity between the overall network and the subtype networks. Conversely, when this condition is not met, some subtypes display gene relationships between i and j, while others do not. Therefore, we decrease the alignment between the overall network and the subtype networks. The tuning parameter *tre* is used to measure the similarity of values between precision matrices.

## 3 Materials and methods

### 3.1 Parameter estimation

The Expectation-Maximization (EM) algorithm (Dempster *et al.* 1977) is employed to obtain the maximum penalized likelihood estimates, as shown in Algorithm 1. We introduce $Z_{lk}$ as the indicator of whether sample l belongs to subtype k. Since $Z_{lk}$ is treated as missing values (Gao *et al.* 2016), we define the posterior probability ${\hat{Z}}_{lk}$ as $P\left(Z_{lk}=1|x_{l},\Theta \right)$, which represents the probability that sample l belongs to subtype k (Tan 2022). The E-step involves updating ${\hat{Z}}_{lk}$ and computing the conditional expectation of the log-likelihood function with penalty:

$$E_{\hat{\Theta }}(\text{log}\;L(\Theta ))=\sum_{l=1}^{n}\sum_{k=1}^{K}{\hat{Z}}_{lk}\;\text{log}\;\left\{\pi_{k}N(x_{l}|\mu_{k},\Omega_{k}{}^{-1})\right\}.$$

 

(7) $$+\sum_{t=1}^{m}\;\text{log}\;\left\{N(x_{t}^{\prime }|\mu_{X},\Omega_{X}{}^{-1})\right\}-P(\Theta ).$$

${\hat{Z}}_{lk}$ is given as:

(8) $${\hat{Z}}_{lk}^{\left(r\right)}=\frac{\pi_{k}^{\left(r-1\right)}N(x_{l}|\mu_{k}^{\left(r-1\right)},\Omega_{k}^{{\left(r-1\right)}^{-1}})}{\sum_{k}\pi_{k}^{\left(r-1\right)}N(x_{l}|\mu_{k}^{\left(r-1\right)},\Omega_{k}^{{\left(r-1\right)}^{-1}})}.$$

Here, r represents the iteration number in the EM algorithm.

In the M-step, maximization with respect to parameter $\mu_{k}$, $\mu_{X}$ and $\pi_{k}$ can be achieved by taking the derivative of the log-likelihood in (7). The updating equations are obtained as follows: ${\hat{\mu }}_{k}^{\left(r+1\right)}=\sum_{l=1}^{n}{\hat{Z}}_{lk}^{\left(r\right)}x_{l}/\sum_{l=1}^{n}{\hat{Z}}_{lk}^{\left(r\right)}$, ${\hat{\mu }}_{X}^{\left(r+1\right)}=\frac{1}{m}\sum_{t=1}^{m}x_{t}^{\prime }$, and $\pi_{k}{}^{\left(r+1\right)}=\frac{1}{n}\sum_{l=1}^{n}{\hat{Z}}_{lk}^{\left(r\right)}$. Ω is updated using the Alternating Direction Method of Multipliers (ADMM) algorithm (Boyd *et al.* 2010), following an approach similar to the JGL estimation (Danaher *et al.* 2014) and its extensions (Lyu *et al.* 2018, Ren *et al.* 2022, Seal *et al.* 2023). Compared to previous methods, our penalty term includes adaptive weights and the overall network, involving more complex network relationships. The specific implementation of the ADMM algorithm for AJGM and the complete process of EM algorithm is provided in Supplementary Section S1.

Algorithm 1 Algorithm for Parameter Estimation in AJGM

- Input: Gene expression data X, overall samples $X^{\prime }$, number of subtype K, tuning parameters $\lambda_{1},\lambda_{2},\lambda_{3}$, γ and *tre*
- Initialization: $\Omega_{k}=\Omega_{X}=I,\;\mu_{k}\;\text{and}\;\pi_{k}$ are obtained from K-means clustering
- Repeat the following steps in the EM algorithm:

1. In the E step, update ${\hat{Z}}_{lk}$
2. In the M-step, update $\mu_{X},\;\mu_{k}\;\text{and}\;\pi_{k}$
3. In the M-step, update the precision matrices using ADMM Algorithm

- Output: Overall network, subtype-specific networks, and samples clustering results

### 3.2 Data imputation

Some gene sequencing data may deviate from a normal distribution, violating the GGM assumptions. For instance, scRNA-seq captures gene expression at a single-cell resolution, but its data is often highly sparse and prone to zero inflation, leading to deviations from a normal distribution. Consequently, imputation methods are necessary to address these deviations and ensure accurate modeling.

Following the CYBERTRACK model (Minoura *et al.* 2021), which performs data imputation within a mixture Gaussian framework, we carry out data imputation at each iteration of the EM algorithm. Assume that sample l belongs to subtype k, then $x_{l}|C_{l}∼N\left(\mu_{k},\Omega_{k}^{-1}\right)$. After determining a sample’s subtype and corresponding parameters $\left(\mu_{k},\Omega_{k}^{-1}\right)$, zero values are imputed by sampling from the subtype’s Gaussian distribution. The feasibility of data imputation, along with the detailed process of the EM algorithm incorporating data imputation, is outlined in Supplementary Section S2.

### 3.3 Tuning parameter selection

The tuning parameters in the model include the number of subtypes K, the penalty parameters $\lambda_{1}$, $\lambda_{2}$, and $\lambda_{3}$, as well as the adaptive weights parameter γ and threshold *tre*. In this study, parameters can be selected using the Bayesian Information Criterion (BIC), and an iterative network search is recommended to optimize these values. Initially, we assign starting values for parameters $\lambda_{1}$, $\lambda_{2}$, $\lambda_{3}$, γ, and *tre*, and use the BIC criterion to determine the optimal K. Subsequently, we update the parameters $\lambda_{1}$, $\lambda_{2}$, $\lambda_{3}$, γ, and *tre* based on the chosen K and recalculate K iteratively. Once K stabilizes, we finalize the parameter set. Detailed instructions for calculating the BIC value and recommendations for parameter selection are provided in Supplementary Section S3.

## 4 Simulation study

### 4.1 Data generation

**Figure 2.**
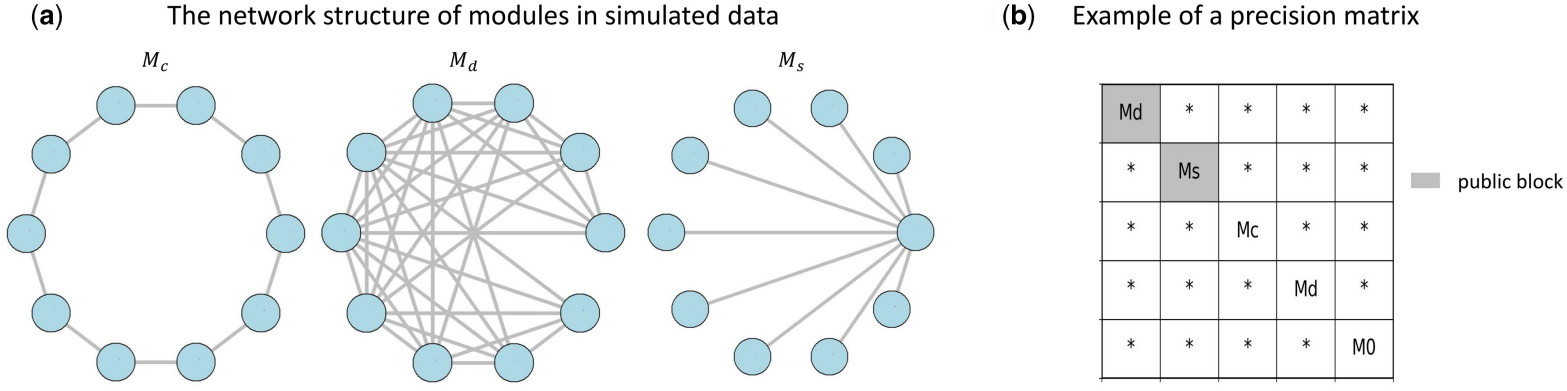
The generation method of simulated data. (a) Network structure for three modules. (b) An example of a precision matrix generated when p=50.

In the simulation study, we generate data from Gaussian distributions to validate the performance of AJGM. The approach for generating precision matrices for the simulated data is based on a modular generation method (Wu and Luo 2022). The precision matrix $\Omega_{k}$ is composed of multiple modules, each of size 10×10. Each module belongs to one of four types (Fig. 2a): $M_{d}$ (dense module), $M_{c}$ (circle module), $M_{s}$ (star module), and $M_{0}$ (zero module, which does not exhibit any genetic relationships).

We provide an example to demonstrate the generation process of the precision matrix for p=50 (Fig. 2b). Each block in $\Omega_{k}$ has a 30% probability of being a public block, which is consistent across all subtype precision matrices, representing shared gene relationships. The module for the public block is randomly selected from $M_{d}$, $M_{c}$, and $M_{s}$. Modules for the non-public blocks are randomly selected from $M_{d}$, $M_{c}$, $M_{s}$, and $M_{0}$. Details of the module values and the mean vector generation process are provided in Supplementary Section S4. In all simulations presented in this article, we generate 30 datasets with p=100 to assess network estimation capability.

### 4.2 Subtype network inference

We select BLGGM (Wu and Luo 2022), GGMPF (Ren *et al.* 2022), MGGM (Tan 2022), SCGGM (Li and Li 2018), and SILGGM (Zhang *et al.* 2018) as comparison methods. The network inference performance is assessed based on False Positive Rate (FPR), True Positive Rate (TPR) and F1 scores, while the sample classification performance is evaluated using adjusted Rand index (ARI). Details regarding the comparative methods and the calculation procedures for the evaluation metrics are presented in Supplementary Sections S5 and S6. Furthermore, in the simulation study, we set the overall samples $X^{\prime }$ as X because this method exhibits relatively high accuracy, as shown in Supplementary Table S2. This difference arises because the sampling-based method produces mimic data derived from X, which may lead to information loss and approximation errors. The network inference accuracy of generating $X^{\prime }$ by sampling is presented in Supplementary Tables S2 and S3.

Data are initially generated with $n_{k}=300$ for K=3 and K=4, and the corresponding results are displayed in Table 1. For K=3, regarding recall and F1, AJGM significantly outperforms other methods. For K=4, recall and F1 scores of all methods show a decrease compared to the K=3 scenario. GGMPF and MGGM demonstrate sample misclassification in certain datasets. AJGM shows superior performance in terms of FPR, TPR, and F1 compared to other methods.

### 4.3 Subtype network inference with zero-inflated data

To simulate the high proportion of observed zeros in scRNA-seq data, we generate zero-inflated data. Initially, the data are generated with $n_{k}=300$ and K=3. Subsequently, a fraction of values is set to zero based on a probability that depends on the original value, as genes with lower expression levels are more likely to go undetected.

We use two methods to introduce zero inflation. In the first method, the probability that $x_{ij}$ is set to zero is given by:

$$p_{ij}=e^{-\alpha x_{ij}},$$

where α is a tuning parameter that controls the proportion of zeros in the dataset. In the second method, the probability that $x_{ij}$ is set to zero is defined as:

$$p_{ij}=1-\beta x_{ij},$$

where β is a tuning parameter that similarly regulates the proportion of zeros.

Among the available methods, only AJGM, BLGGM, SCGGM, and SILGGM are applicable to scRNA-seq data, and these are selected for comparison. As the value of tuning parameter increases, the proportion of zeros decreases, and the estimation accuracy of all approaches improves. Across various values of tuning parameters for two data generation methods, AJGM consistently outperforms comparison methods in both recall and F1 metrics (Supplementary Table S1).

### 4.4 Overall network inference

AJGM identifies shared relationships through the overall network and promotes their appearance in subtype networks during joint estimation. The feasibility of this concept relies on the accuracy of $\Omega_{X}$ in identifying shared relationships. In terms of shared relationship identification, $\Omega_{X}$ shows high recall but relatively low precision values (Supplementary Fig. S2). This occurs primarily because certain gene relationships, present in multiple but not all samples, are occasionally misidentified as being shared across all subtypes in $\Omega_{X}$. Overall, $\Omega_{X}$ performs adequately in identifying shared relationships, as the overall network only needs to increase the probability of potential shared edges appearing in subtype networks, rather than directly estimating these edges. A more detailed discussion of the overall network can be found in Supplementary Section S7.

### 4.5 Shared relationship inference

**Figure 3.**
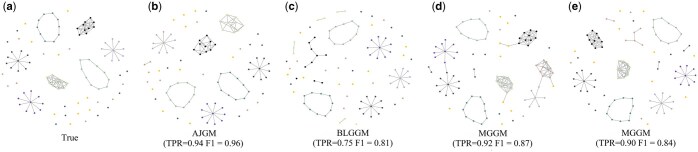
The estimated network for shared relationships of different methods and the true network in simulation study. Subfigure (a) represents the real network, subfigures (b-e) represent the networks estimated by the corresponding methods, with the TPR and F1 scores provided below each subfigure.

In AJGM, we incorporate the overall network to prioritize shared edges, making them more likely to appear in subtype-specific networks. By evaluating the performance of different methods on simulated datasets, we demonstrate that introducing the overall network enhances the ability to estimate shared edges, thereby improving the accuracy of network inference. Metrics are computed separately for public and non-public blocks in simulated data to evaluate AJGM’s capability in inferring shared relationships (Supplementary Table S4). Compared to other competing approaches, AJGM almost accurately estimates all shared relationships (Fig. 3). In addition, we find that the public modules estimated by AJGM exhibit higher accuracy compared to the non-public modules, a trend not observed in the comparative methods. We attribute this phenomenon to the introduction of an overall network $\Omega_{X}$ in joint estimation.

### 4.6 Computational performance

We also evaluate the runtime of AJGM on simulated datasets with varying sample dimensions. For comparison, we include the MGGM method, as both MGGM and AJGM leverage the EM and ADMM algorithms for precision matrix estimation. As illustrated in Supplementary Section S8, AJGM demonstrates a significant computational speed advantage over MGGM across various values of sample dimension, with the advantage becoming increasingly pronounced as the number of genes grows.

## 5 Real application

We apply our method to the scRNA-seq data of triple-negative breast cancer (TNBC). Among breast cancer subtypes, TNBC exhibits the highest level of heterogeneity, characterized by substantial variations in the biological characteristics across different cell types. Consequently, investigating the cellular heterogeneity of TNBC is essential for enhancing treatment outcomes and developing personalized treatment strategies (Nikolski *et al.* 2024). In this section, we will also demonstrate the construction of gene networks using bulk RNA-seq data to show that AJGM is applicable to both single-cell and bulk data.

Scholars have proposed various subtyping methods to address the heterogeneity of TNBC, yet these methods are inadequately integrated and lack a comprehensive approach. Additionally, the relationship between TNBC subtypes and gene networks is not well understood. The heterogeneity of TNBC is reflected in the distinct gene networks of its subtypes, yet most models do not adequately account for this aspect during classification. Using AJGM, we can achieve simultaneous sample classification and network inference in TNBC cells with unknown heterogeneity, allowing for the investigation of both shared and subtype-specific gene relationships.

### 5.1 Data

The data utilized in this study are obtained from single-cell RNA sequencing data of 220 TNBC cells (Chung *et al.* 2017), accessible from the GEO database (ID: GSE75688). We select genes highly correlated with the disease, guided by the PAM50 gene set (Parker *et al.* 2009). Additionally, we reference other research studies (Lyu *et al.* 2018, Tang *et al.* 2018) and incorporate hub genes from known breast cancer gene networks into the analysis. These hub genes are merged with the PAM50 gene set, resulting in a final selection of 79 genes for subsequent investigation. The list of these genes can be found in Supplementary Section S10.

**Figure 4.**
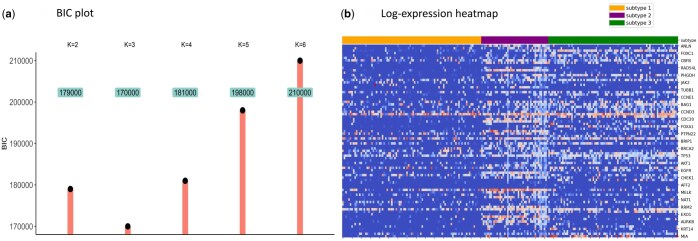
(a) The BIC plot for selecting *K*. (b) The log-expression heatmap for scRNA-seq after cell classification, where darker shades indicate lower expression values and lighter shades indicate higher values.

Given the presence of outliers significantly higher than the mean value in the dataset, we apply a logarithmic transformation to satisfy the assumption of multivariate normality. The pBIC plot indicates the presence of three subtypes (Fig. 4a).

### 5.2 Results of cell classification and network inference

The 220 cell samples are divided into three categories, containing 91, 44, and 85 samples, respectively. Subtype 1 exhibits a higher proportion of zero values, while Subtype 2 overall has higher gene expression values (Fig. 4b). In gene networks, we focus on the shared and specific relationships among subtypes. There are 66 relationships shared among the three subtype networks. The first subtype exhibits 50, the second subtype exhibits 119, and the third subtype exhibits 78 subtype-specific relationships. The complete gene network and cell classification results are demonstrated in Supplementary Tables S5 and S6, Figs S5–S8, and S12.

### 5.3 Biological significance

**Figure 5.**
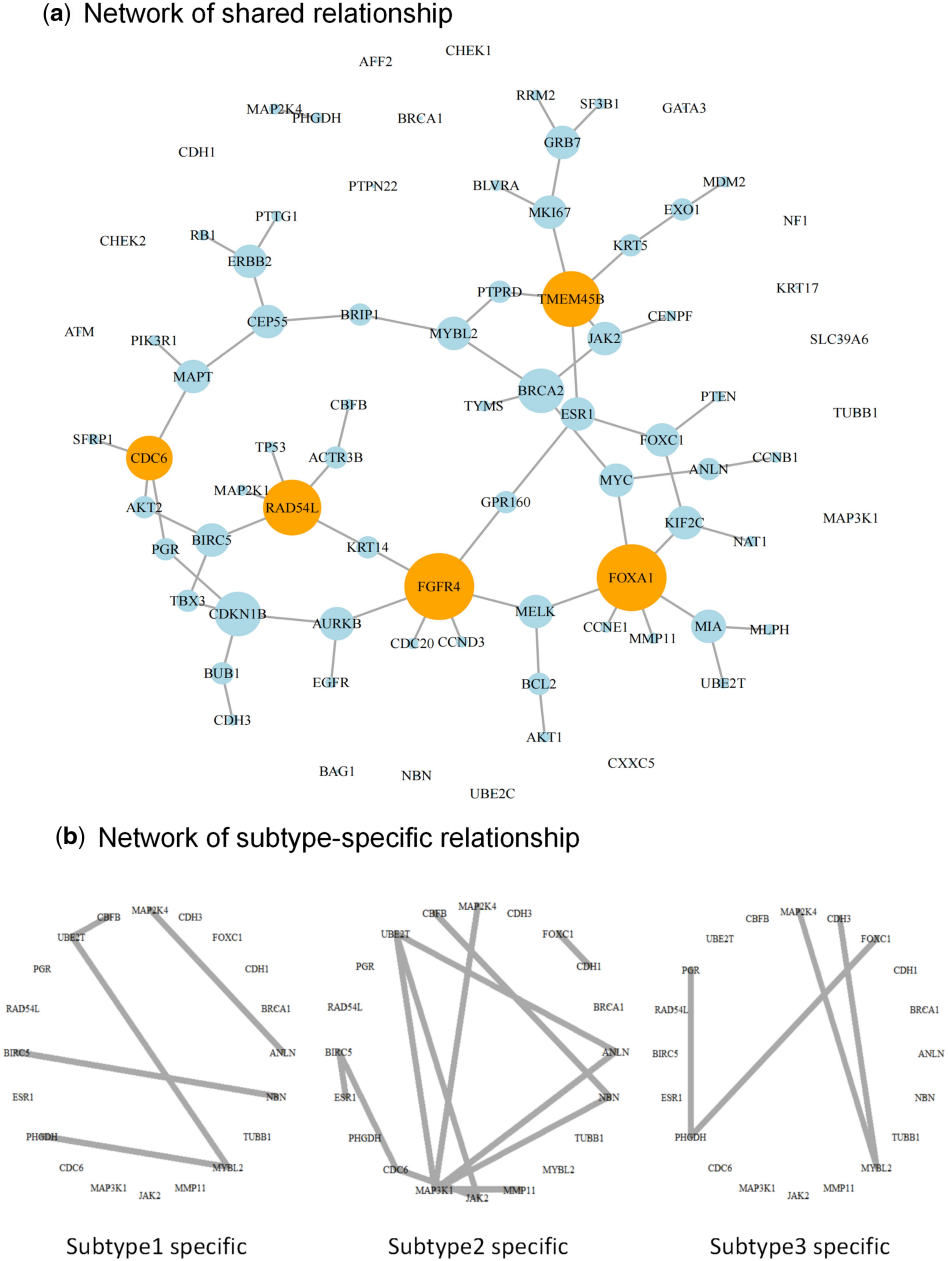
(a) Gene network for relationships shared across subtypes constructed from scRNA-seq of TNBC. (b) Gene networks for subtype-specific relationship constructed from scRNA-seq of TNBC.

We first focus on the shared gene relationships (Fig. 5a). These relationships are present in all subtypes of TNBC, representing common biological features. Notably, FOXA1 exhibits the highest gene degree and is implicated in the positive regulation of the cell cycle pathway (Table 2). Previous studies have suggested that reduced FOXA1 expression may result in malignancy and enhanced cancer stemness, potentially due to disruptions in cell cycle regulation. Moreover, FOXA1 serves as a subtype marker for the identification of TNBC (Dai *et al.* 2019). FGFR4 also exhibits one of the highest degrees. Elevated FGFR4 expression in TNBC is linked to poorer prognostic outcomes, such as reduced survival times, suboptimal chemotherapy responses, and increased lymph node metastasis (Wei *et al.* 2020).

**Table 1.** Performance metrics of subtype networks.

	*K*	AJGM	BLGGM	GGMPF	MGGM	SCGGM	SILGGM
**FPR**	3	**0.01 (0.00)**	**0.01 (0.00)**	0.03 (0.00)	0.04 (0.00)	0.02 (0.00)	**0.01 (0.00)**
	4	**0.01 (0.00)**	**0.01 (0.00)**	0.09 (0.02)	0.02 (0.00)	0.03 (0.00)	**0.01 (0.00)**
**TPR**	3	**0.90 (0.04)**	0.65 (0.12)	0.74 (0.07)	0.76 (0.07)	0.77 (0.03)	0.83 (0.04)
	4	**0.82 (0.06)**	0.53 (0.06)	0.51 (0.20)	0.74 (0.08)	0.72 (0.04)	0.75 (0.05)
**F1**	3	**0.89 (0.03)**	0.76 (0.10)	0.84 (0.04)	0.65 (0.07)	0.80 (0.03)	0.79 (0.02)
	4	**0.77 (0.05)**	0.62 (0.05)	0.61 (0.17)	0.66 (0.07)	0.75 (0.03)	0.76 (0.04)
**ARI**	3	**1.00 (0.00)**	**1.00 (0.00)**	**1.00 (0.00)**	**1.00 (0.00)**	**1.00 (0.00)**	\
	4	**1.00 (0.00)**	**1.00 (0.00)**	0.59 (0.10)	0.93 (0.14)	**1.00 (0.00)**	\

The best results are indicated in bold.

**Table 2.** Hub genes and Go term enrichment.

Network name	Hubs	Go term enrichment	FDR
Overall network	FOXA1, FGFR4	Mitotic cell cycle phase transition	1.40E−20
		Positive regulation of cell cycle	1.84E−11
		Positive regulation of kinase activity	3.65E−05
Subtype1-specific network	CDC20	Cell cycle checkpoint signaling	4.70E−16
Subtype2-specific network	MAP3K1	Regulation of stress-activated MAPK cascade	8.10E−05
Subtype3-specific network	MYC	Signal transduction by p53 class mediator	4.37E−10

We next focus on gene relationships specific to particular subtypes (Fig. 5b). These relationships provide insights into the cellular heterogeneity of TNBC. Subtype 2, among the three subtypes identified by AJGM, has the smallest sample size but the greatest number of gene interrelationships. Dense genetic networks may suggest enhanced or aberrant activation of specific signaling pathways. For instance, tumor growth and metastasis are accelerated by the activation of signaling pathways involved in cell proliferation, invasion, and metastasis. Consequently, subtype 2 is likely the most invasive subtype. Enrichment analysis reveals that a greater number of genes in subtype 2 are associated with pathways involved in cancer cell metastasis and invasion compared to the other subtypes.

The hub genes of the three subtype-specific networks also differ. The hub gene of the first subtype is CDC20. Aberrant expression of CDC20 is associated with premature anaphase promotion, resulting in mitotic abnormalities that may contribute to tumorigenesis. The hub gene of the second subtype is MAP3K1, a key regulator of signaling cascades that control tumor proliferation and metastasis (Sun *et al.* 2015). MAP3K1, a member of the MAPK family, participates in multiple MAPK signaling pathways, as indicated by enrichment analysis, and regulates essential cellular processes related to growth and survival (Kuo *et al.* 2023). The hub gene of the third subtype is MYC. Dysregulation of MYC promotes the accelerated development and metastasis of heterogeneous triple-negative breast tumors, contributing to the emergence of more aggressive subtypes.

Additionally, we utilize two comparative methods, JGL (Danaher *et al.* 2014) and WGCNA (Langfelder and Horvath 2008), for gene network construction. Enrichment analysis reveals that AJGM, with its superior network estimation accuracy, offers more biologically relevant insights. For example, AJGM identifies pathways promoting cell cycle activation and proliferation, such as Cell Cycle Checkpoint Signaling and Regulation of Mitotic Metaphase/Anaphase Transition. In contrast, JGL and WGCNA highlight pathways like Negative Regulation of Cell Cycle and Negative Regulation of Cell Cycle Process, which are less aligned with the aggressive traits of TNBC. By prioritizing shared relationships between subtypes, AJGM effectively captures both shared and distinct features of subtypes, enabling more insightful downstream biological analyses. More detailed information on the downstream biological analysis of TNBC can be found in Supplementary Section S11.

### 5.4 Application on bulk RNA-seq data of TNBC

Models similar to AJGM, capable of simultaneously performing sample clustering and inferring networks, are particularly suited to scRNA-seq data. This capability arises from the model’s ability to utilize single-cell resolution data for precise cell classification. In contrast, bulk RNA-seq represents the average of cell populations, making it impossible to classify subtypes at the single-cell level. However, AJGM can still be applied to bulk RNA-seq data to construct gene networks for different subtypes.

We obtain bulk RNA-seq data for 108 TNBC samples from the TCGA project (The Cancer Genome Atlas Network 2012) and select 79 genes consistent with those studied in the scRNA-seq analysis. AJGM classify the 108 samples into three groups, containing 22, 17, and 69 samples, respectively. In the gene networks, 152 edges are shared among all subtypes. Each subtype has 164, 36, and 91 unique edges, respectively. In the network of shared relationships, genes like FOXA1 and FOXC1 exhibit high degrees, similar to those in the network previously constructed using scRNA-seq data. Detailed network information is provided in Supplementary Tables S7 and S8 and Figs S9–S12, and S14.

### 5.5 Application on time-series sequencing data

We also apply AJGM to time series sequencing data to explore the scalability of our proposed model. The first dataset is scRNA-seq data from Arabidopsis root procambium-type cells (Shahan *et al.* 2022), where all cells are annotated into 10 distinct time points (T0–T10) through related models and manual verification. Based on AJGM, we reclassify the samples into three stages: early, middle, and late stages. Moreover, since cells from time points that are close to each other tend to share more common pathways, we extend AJGM by dynamically adjusting the penalty term in the network order based on the prior time labels in the dataset, ensuring that the network is arranged in chronological order. The clustering results of the cells show high consistency with the dataset’s prior time labels, demonstrating the reliable subclassification capability of AJGM. Additionally, we identify hub genes in the network corresponding to the respective time periods, with early-stage hub genes being more related to growth and development, while those in the middle and late stages are more associated with differentiation. The detailed analysis is provided in Supplementary Section S12.

The second dataset is single-cell RNA sequencing data of mouse embryonic stem cells (Klein *et al.* 2015), used to study the differentiation process regulated by leukemia inhibitory factor (LIF). The dataset is collected at four time points, and we select data from Day 0 (stage 1) and Day 7 (stage 2) to explore hub genes affecting stem cell growth. By examining the network of shared relationships, we can observe which regulatory relationships change across all cells in heterogeneous cell samples at different time points. The results indicate that LIF regulates stem cell pluripotency and inhibits differentiation, as shown by the reduced degree of genes related to differentiation, such as LEFTY2, CDX2, and COL1A1, at Day 7. Detailed information about this real application can be found in Supplementary Section S13.

## 6 Discussion

We present a method named AJGM for estimating gene networks from data exhibiting unknown heterogeneity. zCompared to existing methods, AJGM offers several advantages. Firstly, an overall network is introduced in joint estimation, with relationships shared by all samples being accurately captured, thus enhancing the entire estimation accuracy of the network. Secondly, it utilizes adaptive weights to automatically adjust similarity in network structures across networks, addressing theoretical limitations of existing methods as relationships between different gene pairs may vary across subtypes. Moreover, AJGM can handle zero-inflated scRNA-seq data through data imputation, further extending the applicability of GGMs. Our AJGM consistently outperforms existing methods in network inference and sample classification on synthetic datasets, across datasets with varying numbers of subtypes and datasets exhibiting zero-inflation.

There are several directions to improve the current work. Although the reliability of data imputation method in AJGM has been demonstrated, applying this method to single-cell data can be challenging when a high proportion of zeros is present. This challenge arises because, during each iteration of the EM algorithm, even after parameter convergence, replacing zeros with sampled values can modify the dataset, requiring re-estimation of the parameters. To address this issue, employing a sampling method with robust convergence properties, such as Gibbs sampling, may prove effective.

In addition, the method for estimating the overall network can be improved. Theoretically, the overall network should only contain gene relationships present in all samples. However, it may also include relationships that are found in the majority of samples but are not necessarily present in all subtypes. One approach to addressing this issue can be separately estimate common and non-common parts of subtype networks (Wu *et al.* 2020). This method can yield an overall network that is more valuable in practical applications.

An important direction for future research involves enhancing AJGM to support higher-dimensional network estimation. For instance, we are exploring its application to spatial transcriptomics data, enabling the joint estimation of gene networks across spatially adjacent cell subtypes. Furthermore, we aim to extend AJGM to temporal sequencing datasets to capture dynamic regulatory relationships over time. A more ambitious endeavor would be the integration of spatial and temporal dimensions into a unified three-dimensional framework. These developments highlight the remarkable scalability and adaptability of AJGM for analyzing increasingly complex biological data.

## Supplementary Material

btaf096_Supplementary_Data

## Data Availability

The data underlying this article are available in public repositories as follows: The single-cell RNA sequencing data of triple-negative breast cancer are available in the Gene Expression Omnibus (GEO) with accession number GSE75688. The single-cell RNA sequencing data from Arabidopsis root procambium-type cells are available in GEO with accession number GSE152766. The single-cell RNA sequencing data of mouse embryonic stem cells are available in GEO with accession number GSE65525. The bulk RNA sequencing data of triple-negative breast cancer are available in The Cancer Genome Atlas (TCGA) at https://cancergenome.nih.gov/.
