# Supplementary material for: AJGM: joint learning of heterogeneous gene networks with adaptive graphical model
Source: Bioinformatics. 2025 Mar 12;41(3):btaf096. doi: 10.1093/bioinformatics/btaf096 (PMC11937957; doi:10.1093/bioinformatics/btaf096)
Supplement: btaf096_Supplementary_Data [file btaf096_supplementary_data.zip › Bioinformatics_supplement_ final.pdf]

Supplementary Materials for Adaptive Joint Graphical Model (AJGM): Joint Learning of Multiple Gene Networks with Adaptive Weights

Shunqi Yang[1], Lingyi Hu[1], Pengzhou Chen[1], Xiangxiang Zeng[2], and Shanjun Mao[1,*]

[1]Department of Statistics, Hunan University, Changsha 410000, China
[2]College of Computer Science and Electronic Engineering, Hunan University, Changsha 410000, China
[*]Corresponding author: shjmao@hnu.edu.cn

List of Supplementary Materials

- Section S1. The complete process of the EM algorithm and the ADMM algorithm.
- Section S2. The parameter inference process involving data imputation.
- Section S3. Details on calculating the BIC value and suggestions on selecting parameters.
- Section S4. Data generation method in simulation study.
- Section S5. Details of implementing competing methods in Simulation Study.
- Section S6. Details of evaluation metrics in Simulation Study.
- Section S7. Simulation study on the overall network.
- Section S8. Computational performance analysis of proposed model.
- Section S9. Simulation study on the impact of precision matrix order on the accuracy of network estimation.
- Section S10. List of 79 genes used in real application.
- Section SS11. Downstream biological analysis for TNBC in Section Real Applications.

- Section S12. Real application on application on Arabidopsis root sequencing data.
- Section S13. Real application on sequencing data of mouse embryonic stem cells.
- Figure S1: The estimated precision matrices by AJGM and the true precision matrices in simulation study when $p = 100$ and $K = 3$.
- Figure S2: Precision, recall and F1 of the estimated overall network in simulation study.
- Figure S3: Keeping other parameters constant, the network's inference precision, recall, and F1 score vary with different values of γ in simulation study.
- Figure S4: Keeping other parameters constant, the network's inference precision, recall, and F1 score vary with different values of λ_3 in simulation study.
- Figure S5: The gene network of shared relationships in triple-negative breast cancer constructed using scRNA-seq data.
- Figure S6: The subtype 1 specific network of triple-negative breast cancer constructed using scRNA-seq data.
- Figure S7: The subtype 2 specific network of triple-negative breast cancer constructed using scRNA-seq data.
- Figure S8: The subtype 3 specific network of triple-negative breast cancer constructed using scRNA-seq data.
- Figure S9: The network of shared relationships in triple-negative breast cancer constructed using Bulk RNA-seq data.
- Figure S10: The subtype 1 specific network of triple-negative breast cancer constructed using Bulk RNA-seq data.
- Figure S11: The subtype 2 specific network of triple-negative breast cancer constructed using Bulk RNA-seq data.
- Figure S12: The subtype 3 specific network of triple-negative breast cancer constructed using Bulk RNA-seq data.
- Figure S13: Number of shared edges and specific edges among different subtypes of gene networks constructed using single-cell RNA-seq data.
- Figure S14: Number of shared edges and specific edges among different subtypes of gene networks constructed using Bulk RNA-seq data.
- Figure S15: The histograms of scRNA-seq and imputed data for 9 genes in the real application of TNBC.

- Figure S16: Proportions of different types of relationships in gene networks constructed from scRNA-seq data of triple-negative breast cancer using AJGM and two comparative methods.
- Table S1. Performance metrics of zero-inflated data in simulation study.
- Table S2. Performance metrics of models for two overall sample generation methods.
- Table S3. Performance metrics of obtaining $x_l^{'}$ from sampling with zero-inflated data in simulation study.
- Table S4. Performance metrics of public and nonpublic blocks.
- Table S5. The hub genes in subtype shared network of triple-negative breast cancer constructed using scRNA-seq data.
- Table S6. The hub genes in the triple-negative breast cancer subtype specific networks constructed using scRNA-seq data.
- Table S7. The hub genes in subtype shared network of triple-negative breast cancer constructed using Bulk RNA-seq data.
- Table S8. The hub genes in the triple-negative breast cancer subtype specific network constructed using Bulk RNA-seq data.

S1. The complete process of the EM algorithm and the ADMM algorithm.

We use EM algorithm to estimate model parameters. In the algorithm, the posterior probability $\hat{Z}_{lk}$ which is defined as $\hat{Z}_{lk} = P\left(\hat{Z}_{lk}|x_l, \Theta\right)$ indicats the probability that sample l belongs to subtype k . In the E-step, the conditional expectation of the log-likelihood function with penalties is,

$$E_{\hat{\Theta}}(\log L(\Theta)) = \sum_{l=1}^{n}\sum_{k=1}^{K}\hat{Z}_{lk}\log\left\{\pi_k N(x_l|\mu_k, {\Omega_k}^{-1})\right\} + \sum_{t=1}^{m}\log\left\{N(x_t^{'}|\mu_X, {\Omega_X}^{-1})\right\} - P(\Theta)$$

Posterior probability $\hat{Z}_{lk}$ is given as,

$$\hat{Z}_{lk}^{(r)} = \frac{\pi_k^{(r-1)}N(x_l|\mu_k^{(r-1)}, {\Omega_k^{(r-1)}}^{-1})}{\sum_k \pi_k^{(r-1)}N(x_l|\mu_k^{(r-1)}, {\Omega_k^{(r-1)}}^{-1})}$$

Here, r represents the current iteration number in the EM algorithm, $\pi_k^{(r-1)}$, $\mu_k^{(r-1)}$, $\Omega_k^{(r-1)}$are the parameters obtained from the M-step in the previous iteration.

In the M-step, the estimation of parameters can be achieved by maximizing the log-likelihood function. We rewrite the equation as

$$\begin{aligned} E_{\hat{\Theta}}(\log L(\Theta)) = &\sum_{l=1}^{n}\sum_{k=1}^{K}\hat{Z}_{lk}\left[\log\pi_k + \frac{1}{2}\log\det\left(\Omega_k\right) - \frac{1}{2}(x_l-\mu_k)^T\Omega_k\left(x_l-\mu_k\right)\right] \\ &+ \sum_{l=1}^{m}\left[\frac{1}{2}\log\det\left(\Omega_X\right) - \frac{1}{2}\left({x_l}^{'}-\mu_X\right)^T\Omega_X\left({x_l}^{'}-\mu_X\right)\right] - P(\Theta) \end{aligned}$$

Taking μ_k as an example and ignoring terms without μ_k, the log-likelihood function regarding μ_k can be derived as,

$$L(\mu_k) = \sum_{l=1}^{n}\sum_{k=1}^{K}\hat{Z}_{lk}^{(r)}\left[-(x_l-\mu_k)^T\Omega_k\left(x_l-\mu_k\right)\right]$$

By taking the partial derivative of $L(\mu_k)$ with respect to μ_k, the updating equation for μ_k in the current iteration is obtained,

$$\hat{\mu}_k^{(r+1)} = \frac{\sum\limits_{l=1}^{n}\hat{Z}_{lk}^{(r)}x_l}{\sum\limits_{l=1}^{n}\hat{Z}_{lk}^{(r)}}$$

Similarly, other parameters can be solved using the same method. The updating equation for μ_X is,

$$\hat{\mu}_X^{(r+1)} = \frac{1}{m}\sum_{l=1}^{m}x_l^{'}$$

The updating equation for π_k is,

$$\pi_k{}^{(r+1)} = \frac{1}{n}\sum_{l=1}^{n}\hat{Z}_{lk}^{(r)}$$

When updating the precision matrix Ω, only terms containing Ω_k and Ω_X are considered,

$$\begin{aligned}L\left(\Omega_k,\Omega_X\right) = &\sum_{l=1}^{n}\sum_{k=1}^{K}\hat{Z}_{lk}\left[\frac{1}{2}\log\det\left(\Omega_k\right) - \frac{1}{2}(x_l-\mu_k)^T\Omega_k\left(x_l-\mu_k\right)\right]\\ &+\sum_{l=1}^{m}\left[\frac{1}{2}\log\det\left(\Omega_X\right) - \frac{1}{2}\left(x_l{}' - \mu_X\right)^T\Omega_X\left(x_l{}'-\mu_X\right)\right] - P(\Theta)\end{aligned} \tag{1}$$

Let $\tilde{S}_k^r = \frac{\sum\limits_{l=1}\hat{Z}_{lk}(x_l-\mu_k)^T(x_l-\mu_k)}{\sum\limits_{l=1}\hat{Z}_{lk}}$, $S_X = \frac{1}{n}\sum\limits_{l=1}\left(x_l^{'}-\mu_X\right)^T\left(x_l^{'}-\mu_X\right)$, $\sum\limits_{l=1}^{n}\hat{Z}_{lk}^{(r)} = n_k$, $\tilde{S}_k^r = S_k$ then Equation(1) can be written as,

$$\begin{aligned}L\left(\Omega_k,\Omega_X\right) = &\frac{1}{2}\sum_{k=1}^{K}n_k\left[\log\det\left(\Omega_k\right) - tr\left(S_k\Omega_k\right)\right]\\ &+\frac{1}{2}m\left[\log\det\left(\Omega_X\right)-tr\left(S_X\Omega_X\right)\right] - \lambda_1\left(\sum_{i\neq j}\sum_{k=1}^{K}\theta_{ij}^{(k)} + \sum_{i\neq j}\theta_{ij}^{(X)}\right)\\ &-\lambda_2\sum_{i\neq j}\sum_{k=1}^{K-1}w_{ij}^{(k,k+1)}\left|\theta_{ij}^{(k)}-\theta_{ij}^{(k+1)}\right| - \lambda_3\sum_{i\neq j}f(\theta_{ij}^{(1)},....,\theta_{ij}^{(k)})w_{ij}^{(K,X)}\left|\theta_{ij}^{(K)}-\theta_{ij}^{(X)}\right|\end{aligned} \tag{2}$$

The penalized log-likelihood can be maximized using the ADMM algorithm. The algorithm requires several intermediate variables such as Z, U that do not have any direct interpretation[1]. For convenience, we suppose $\Omega_{K+1} = \Omega_X$, $n_{K+1} = m$ and $S_{K+1} = S_X$. Then objective function can be rewritten as,

$$\underset{\Theta,Z}{\text{minimize}} \quad -\sum_{k=1}^{K+1}n_k\left[\log\det\left(\Omega_k\right) - tr\left(S_k\Omega_k\right)\right] + P\left(Z\right)$$

In this rewritten equation, Ω_X and Ω_k are subject to positive definiteness constraints, and $Z = \{Z_1,...,Z_{K+1}\}$ is subject to the constraint $Z_k = \Omega_k$.
The corresponding scaled augmented Lagrangian is given by,

$$\begin{aligned}L_\rho\left(\Omega,Z,U\right) = &-\sum_{k\ =1}^{K+1}n_k\left[\log\det\left(\Omega_k\right)-tr\left(S_k\Omega_k\right)\right] + P\left(Z\right)\\ &+\frac{\rho}{2}\sum_{k\ =1}^{K+1}\left\|\Omega_k - Z_k + U_k\right\|_F^2 - \frac{\rho}{2}\sum_{k\ =1}^{K+1}\left\|U_k\right\|_F^2\end{aligned} \tag{3}$$

where $U = \{U_1, ..., U_{K+1}\}$ are dual variables and ρ is the non-negative penalty parameter. The solution to the optimization problem (3) is provided in Algorithm 1.

Algorithm 1: Solving the optimization problem (3)

- Input: Penalty parameter ρ (the default value is 1)
- Initialization: $\Omega_k = I, Z_k = 0, U_k = 0, k = (1, ..., K+1)$
- Repeat the following steps:

1. For $k = 1, ..., K+1$ and define r as current iteration number, then minimize Ω_k in Equation (4)

$$-n_k \left[\log\det(\Omega_k) - tr(S_k \Omega_k)\right] + \frac{\rho}{2} \left\| \Omega_k - Z_k^{(r-1)} + U_k^{(r-1)} \right\|_F^2 \tag{4}$$

2. For $k = 1, ..., K+1$, then minimize Z_k in Equation (5)

$$P(Z) + \frac{\rho}{2} \sum_{k=1}^{K+1} \left\| \Omega_k - Z_k + U_{k-1} \right\|_F^2 \tag{5}$$

3. For $k = 1, ..., K+1$ update U_k according to Equation (6)

$$U_k^r = U_k^{r-1} + \Omega_k^r - Z_k^r \tag{6}$$

4. If the condition in Equation below is satisfied, consider the algorithm to have converged and terminate the iteration,

$$\sum_{k=1}^{K+1} \left\| \Omega_k^r - \Omega_k^{r-1} \right\|_1 / \sum_{k=1}^{K+1} \left\| \Omega_k^{r-1} \right\|_1 < 10^{-5}$$

When minimizing Ω_k in Equation(4), the solution can be obtained through eigen-decomposition[2]. Let VDV^T represents the eigen-decomposition of $S_k - \rho/n_k \left(Z_k^{(r-1)} + U_k^{(r-1)} \right)$, where $\tilde{D}_{jj}$ is a diagonal matrix with the j-th diagonal element being

$$\rho/n_k \left(-D_{jj} + \sqrt{D_{jj}^2 + 4\rho/n_k} \right)$$

When minimizing Z_k,we rewrite Equation(5) as,

$$P(\mathrm{Z}) + \frac{\rho}{2} \sum_{k=1}^{K+1} \left\| \mathrm{Z}_k - \mathrm{A}_k \right\|_F^2;\ \mathrm{A}_k = \ \Omega_k^r + \mathrm{U}_k^{r-1}$$

Since the above equation is separable[1], for each pair of matrix elements (i, j),

its minimization problem is,

$$\lambda_1 \sum_{k=1}^{K+1} |\mathrm{Z}_{ij,k}| + \lambda_2 \sum_{k=1}^{K} h_{ij,k} |\mathrm{Z}_{ij,k} - \mathrm{Z}_{ij,k+1}| + \frac{\rho}{2} \sum_{k=1}^{K+1} |\mathrm{Z}_{ij,k} - \mathrm{A}_{ij,k}|^2$$

where $h_{ij,k}$ is the adaptive weights (combining $w_{ij}^{(k,k+1)}$ and $f(\theta_{ij}^{(1)}, ..., \theta_{ij}^{(k)})$).
This problem is known as the weighted one-dimensional fused lasso signal approximator[3], and it can be solved using the `tv1w_1d` function in the `prox_tv` library in Python[1].

The final algorithm flow is as follows. First, initialize θ. Use K-means clustering to divide samples into K classes, and initialize other parameters based on the clustering results, where $(\mu_1, ..., \mu_K, \mu_X)$ are the mean vectors of each class after clustering, μ_X is the mean vector of the representative samples, and $\pi_K = \frac{n_k}{n}$ is the proportion of each class in the total number of samples.
Then, enter the iterative loop of the EM algorithm. In the E-step, update latent variables $\hat{Z}$ using the parameters from the previous iteration. In the M-step, update parameters using $\hat{Z}$, and enter the next iteration loop. Repeat the expectation step and maximization step until convergence of parameters. In the code, convergence is considered when the absolute difference between the current likelihood function and the likelihood function from the previous iteration is less than 10^{-3}. Finally, output precision matrix $(\Omega_1, ..., \Omega_K, \Omega_X)$ and clustering result $C_l = \arg\max_k \left\{\hat{Z}_{l1}, ..., \hat{Z}_{lK}\right\}$.

S2. Explanation of the data imputation and the parameter inference process involving data imputation.

Single-cell RNA sequencing (scRNA-seq) data often exhibit an abundance of zeros due to technical dropout events and low expression levels. As the ordinary multivariate Gaussian model is inadequate for fitting such zero-inflated data, we assume that each sample is drawn from a zero-inflated multivariate Gaussian mixture distribution characterized by the parameter vector μ_k and the precision matrix Ω_k, which represent the mean and covariance structure of cluster k. For samples belonging to subtype k, they follow a zero-inflated multivariate normal distribution.

$$f_{\mathbf{X}}(\mathbf{x}) = p_k \cdot \delta(\mathbf{x}) + (1 - p_k) \cdot f_{\mathbf{Y}}(\mathbf{x}),$$

$$f_{\mathbf{Y}}(\mathbf{x}) = \frac{|\boldsymbol{\Omega}_k|^{1/2}}{(2\pi)^{d/2}} \exp\left(-\frac{1}{2}(\mathbf{x} - \boldsymbol{\mu}_k)^\top \boldsymbol{\Omega}_k (\mathbf{x} - \boldsymbol{\mu}_k) \right),$$

Here, $\delta(\mathbf{x})$ represents the probability mass at zero, $\mathbf{X}$ denotes the sequencing data, $\mathbf{Y}$ denotes the true sequencing data without dropout, and p_k represents the zero-inflation probability in subtype k. In the data imputation step, for samples belonging to subclass k, we replace the zero values in the sequencing data with values sampled from $\left(\mu_k, \Omega_k^{-1}\right)$.

When $\mathbf{X} \neq 0$, the data remains in the original multivariate normal distribution $N(\boldsymbol{\mu}_k, \boldsymbol{\Omega}_k^{-1})$. When $\mathbf{X} = 0$, the imputed data $\tilde{\mathbf{Y}} \sim N(\hat{\boldsymbol{\mu}}_k, \hat{\boldsymbol{\Omega}}_k^{-1})$.

Thus, the distribution of the imputed data $\mathbf{X}'$ can be viewed as a weighted combination of two identical multivariate normal distributions:

$$\mathbf{X}' \sim \pi_k \cdot N(\boldsymbol{\mu}_k, \boldsymbol{\Omega}_k^{-1}) + (1 - \pi_k) \cdot N(\hat{\boldsymbol{\mu}}_k, \hat{\boldsymbol{\Omega}}_k^{-1}).$$

As the algorithm iterates, the estimated $\hat{\boldsymbol{\mu}}_k$ and $\hat{\boldsymbol{\Omega}}_k^{-1}$ will approach the true values $\boldsymbol{\mu}_k$ and $\boldsymbol{\Omega}_k^{-1}$. After the algorithm converges, the imputed data will follow a normal distribution.

The parameter inference process for scRNA-seq, including data imputation, is outlined in Algorithm 2:

Algorithm 2: Algorithm for parameter estimation with data imputation

- Input: Gene expression data x, representative sample $x^{'}$, number of subtypes K, tuning parameters $\lambda_1, \lambda_2, \lambda_3$, γ and tre

- Initialization: $\Omega_k = \Omega_X = I, \mu_k$ and π_k are obtained from K-means clustering results

- Repeat the following steps in EM algorithm:

1. In the E step, update $\hat{Z}_{lk}$

2. In the M-step, update μ_X, μ_k and π_k

3. In the M-step, update the precision matrices using ADMM Algorithm

4. Replace 0 in x by sampling. The subtype membership of samples is determined by $\hat{Z}_{lk}$. The mean vector and precision matrix of the sampled normal distribution are μ_k and Ω_k.

5. If $x^{'}$ is set as x, then directly use imputed x in step 4 as $x^{'}$. If $x^{'}$ is generating by sampling, then utilize the imputed x to re-estimate the parameters of the sampling distribution (mean vector and precision matrix of the normal distribution) and regenerate $x^{'}$.

6. If the absolute difference between the likelihood functions of the current and previous iterations is less than 0.001, EM algorithm is considered to have converged, and the iterations are stopped.

- Output: Overall gene network, subtype-specific gene networks, and subtype clustering results

S3. Details on calculating the BIC value and suggestions on selecting parameters.

The formula of BIC is

$$BIC\left(K,\lambda_1,\lambda_2,\lambda_3,\gamma\right)=-2\log\left(L\left(\left(\hat{\pi}_k,\hat{\mu}_k,\hat{\Omega}_k\right),k=1,...,K|X\right)L\left(\left(\hat{\mu}_X,\hat{\Omega}_X\right)|X'\right)\right)$$
$$+\log\left(N\right)d$$

N represents the number of samples in the model, which is the sum of the samples for x_l and $x_l^{'}$. d is the number of parameters in the model and equals

$$K-1+\left(K+1\right)p+\left(K+1\right)p\left(p+1\right)/2$$

We recommend using grid search for parameter selection. However, using the criterion like BIC for grid search may not always yield optimal parameters. Network estimation is usually used for auxiliary data exploration, and the use of criterion like BIC may tend to select models with parameters that are too large to be useful. Therefore, model selection should be based on practicality, including good model interpretability and stable estimation results. We recommend establishing a rough range of parameters before using the model to ensure reasonable results, and then proceeding with grid search. In addition, during grid search, always ensure that parameter λ_3 is greater than λ_2 to satisfy the model assumptions.

S4. Data generation method in simulation study.

In the simulation study, data from each subtype is sampled from different normal distributions. The precision matrix Ω_k consists of 10 modules, each of size 10×10. Each module is one of the following four modules: M_d: dense module, M_c: circle module, M_s: star module, and M_0: zero module. The specific values of four modules are shown in Figure 1.

M_0

2.0	0	0	0	0	0	0	0	0	0
0	2.0	0	0	0	0	0	0	0	0
0	0	2.0	0	0	0	0	0	0	0
0	0	0	2.0	0	0	0	0	0	0
0	0	0	0	2.0	0	0	0	0	0
0	0	0	0	0	2.0	0	0	0	0
0	0	0	0	0	0	2.0	0	0	0
0	0	0	0	0	0	0	2.0	0	0
0	0	0	0	0	0	0	0	2.0	0
0	0	0	0	0	0	0	0	0	2.0

M_c

2	0.9	0	0	0	0	0	0	0	0
0.9	2	0.9	0	0	0	0	0	0	0
0	0.9	2	0.9	0	0	0	0	0	0
0	0	0.9	2	0.9	0	0	0	0	0
0	0	0	0.9	2	0.9	0	0	0	0
0	0	0	0	0.9	2	0.9	0	0	0
0	0	0	0	0	0.9	2	0.9	0	0
0	0	0	0	0	0	0.9	2	0.9	0
0	0	0	0	0	0	0	0.9	2	0.9
0	0	0	0	0	0	0	0	0.9	2

M_d

2	0.7	0.7	0.7	0.7	0.7	0	0	0	0
0.7	2	0.7	0.7	0.7	0.7	0.7	0	0	0
0.7	0.7	2	0.7	0.7	0.7	0.7	0.7	0	0
0.7	0.7	0.7	2	0.7	0.7	0.7	0.7	0.7	0
0.7	0.7	0.7	0.7	2	0.7	0.7	0.7	0.7	0.7
0.7	0.7	0.7	0.7	0.7	2	0.7	0.7	0.7	0.7
0	0.7	0.7	0.7	0.7	0.7	2	0.7	0.7	0.7
0	0	0.7	0.7	0.7	0.7	0.7	2	0.7	0.7
0	0	0	0.7	0.7	0.7	0.7	0.7	2	0.7
0	0	0	0	0.7	0.7	0.7	0.7	0.7	2

M_s

2.0	0.6	0.6	0.6	0.6	0.6	0.6	0.6	0.6	0.6
0.6	2	0	0	0	0	0	0	0	0
0.6	0	2	0	0	0	0	0	0	0
0.6	0	0	2	0	0	0	0	0	0
0.6	0	0	0	2	0	0	0	0	0
0.6	0	0	0	0	2	0	0	0	0
0.6	0	0	0	0	0	2	0	0	0
0.6	0	0	0	0	0	0	2	0	0
0.6	0	0	0	0	0	0	0	2	0
0.6	0	0	0	0	0	0	0	0	2

Figure 1: The specific values of four modules.

Mean vectors for simulated data are sampled from both gamma and normal distributions. For subtype 1, the mean vector is sampled from $\Gamma(5,2)$. For subtype 2, the first 10 elements of the mean vector are sampled from $\mathcal{N}(4,0.04)$, the next 10 elements from $\mathcal{N}(5,0.04)$, while the remaining elements are consistent with subtype 1. For subtype 3, elements 16 to 30 of the mean vector

are sampled from $\mathcal{N}(6, 0.04)$, while the other elements remain consistent with subtype 1. Subtype 4's mean vector is sampled from $\mathcal{N}(8, 0.04)$.

S5. Details of implementing competing methods in Simulation Study.

We compare our AJGM with the following three methods that enable both cell subtype delineation and network inference:

BLGGM: The BLGGM algorithm[4] constructs Bayesian mixture Gaussian graphical models to jointly estimate multiple gene networks. This method takes into account both cellular heterogeneity and data zero-inflation and is applicable to expression data from single cells. The R package is available on `https://github.com/WgitU/BLGGM`.

MGGM: The MGGM algorithm[5] constructs mixture Gaussian graph model and inferring parameters using EM algorithm and MGGM algorithm. The method takes into account the similarity and sparsity of the subtype network in the penalty term and avoids excessive bias through a truncated lasso penalty. In the original paper the authors did not name this method, and for the convenience of expression, this method is uniformly referred to as the MGGM algorithm.

GGMPF: The GGMPF algorithm[6] utilizes the mean vector and precision matrix to construct the penalty function when inferring the parameters of GGM, making the results interpretable and reliable. The R package is available on `https://CRAN.R-project.org/ package=HeteroGGM`.

SCGGM: SCGGM is an algorithm for joint estimation of multiple precision matrices, which incorporates both the sparsity and similarity of the matrices in its penalty term[7]. Additionally, this method adapts a data imputation algorithm[8] designed for zero-inflated data, making it suitable for both scRNA-seq and bulk RNA-seq datasets.

SILGGM: The SILGGM algorithm[9] constructs a Sparse Inverse Covariance Graphical Gaussian Model to efficiently infer large-scale gene networks. This method incorporates sparse regularization to handle high-dimensional data and zero-inflation by applying a customized penalty function that effectively accounts for the abundance of zero values in the data.

Regarding parameter selection for the comparison methods, no parameters needed to be set for BLGGM. The parameters in MGGM and GGMPF were selected using a combination of grid search and BIC criterion, with the grid search range using the defaults from the code.

S6. Details of evaluation metrics in Simulation Study.

We introduce four measurements to evaluate the accuracy of precision matrix: FPR, Precision, Recall(TPR) and F1. Let $\hat{\theta}_{ij}^{(k)}$ be the (i, j) element of the estimated precision matrix for the kth subtype, $\theta_{ij}^{(k)}$ be the (i, j) element of the true precision matrix. Precision, recall and F1 are computed as follows :

$$\mathrm{FPR} = \frac{1}{K}\sum_{k=1}^{K}\frac{\sum_{i<j} 1\left(\hat{\theta}_{ij}^{(k)} > 0, \theta_{ij}^{(k)} = 0\right)}{\sum_{i<j} 1\left(\theta_{ij}^{(k)} = 0\right)} \tag{7}$$

$$\mathrm{Precision} = \frac{1}{K}\sum_{k=1}^{K}\frac{\sum_{i<j} 1\left(\hat{\theta}_{ij}^{(k)} > 0, \theta_{ij}^{(k)} > 0\right)}{\sum_{i<j} 1\left(\hat{\theta}_{ij}^{(k)} > 0\right)} \tag{8}$$

$$\mathrm{Recall} = \frac{1}{K}\sum_{k=1}^{K}\frac{\sum_{i<j} 1\left(\hat{\theta}_{ij}^{(k)} > 0, \theta_{ij}^{(k)} > 0\right)}{\sum_{i<j} 1\left(\theta_{ij}^{(k)} > 0\right)} \tag{9}$$

$$F1 = \frac{2 \times \mathrm{Precision} \times \mathrm{Recall}}{\mathrm{Precision} + \mathrm{Recall}} \tag{10}$$

We introduce ARI to evaluate the accuracy of subtype classification

$$ARI = \frac{\sum_{ij}\binom{n_{ij}}{2} - \left[\sum_i \binom{a_i}{2}\sum_j \binom{b_j}{2}\right]/\binom{n}{2}}{\frac{1}{2}\left[\sum_i \binom{a_i}{2} + \sum_j \binom{b_j}{2}\right] - \left[\sum_i \binom{a_i}{2}\sum_j \binom{b_j}{2}\right]/\binom{n}{2}} \tag{11}$$

In the formula, n_{ij} represents the value in the ith row and jth column of the contingency table, a_i represents the sum of the ith row in the contingency table, and b_i represents the sum of the jth column in the contingency table.

S7. Simulation study on the overall network.

In this section, we focus on the accuracy of estimating the shared relationships in the overall network. The data generation process is consistent with that described in Section S4 of the Supplementary Material. We generated datasets with the number of subtypes ranging from 3 to 6. As the number of subtypes increases, the recall rate of the overall network remains high, while precision slightly decreases. This aligns with our argument in the paper that the overall network captures potential shared relationships but may also include some relationships that are not shared by all samples.

Additionally, we evaluated the estimation accuracy of the subtype networks for different values of k. As shown in Table 1, the network estimates perform well in terms of both recall and precision. Therefore, the union of the edges from all subtype networks results in higher precision compared to the overall network. Additionally, we compute the case where the tuning parameter $\lambda_3 = 0$, in which the overall network does not influence the estimation of the network of shared relationships. Without the support of the overall network, potential shared relationships are less likely to appear in subtype networks, leading to a certain degree of decline in estimation accuracy.

This simulation study illustrates the principle of learning shared relationships in the AJGM model: the overall network identifies potential shared relationships, while the penalty term encourages these edges to appear more frequently in the subtype networks. The subtype networks, in turn, apply an adaptive penalty term to filter out edges in the overall network that are not shared by all subtypes, preventing such relationships from influencing the subtype networks.

Table 1: Performance metrics of estimated networks.

	k	overall network	shred relationship	shred relationship ($\lambda_3 = 0$)
Precision	3	0.791	0.878	0.835
	4	0.825	0.863	0.799
	5	0.737	0.877	0.750
	6	0.694	0.786	0.713
Recall	3	0.91	0.946	0.901
	4	0.901	0.912	0.848
	5	0.8444	0.877	0.832
	6	0.883	0.862	0.814
F1	3	0.847	0.917	0.867
	4	0.813	0.887	0.823
	5	0.787	0.877	0.789
	6	0.778	0.822	0.760

S8. Computational performance analysis of proposed model.

To evaluate the computational performance of our proposed method, we conduct experiments on networks with varying numbers of genes. The experiments are performed on a computer (Apple M1, 8 GB RAM).

Additionally, the MGGM method is used for comparison, as both MGGM and AJGM leverage the EM algorithm and the ADMM algorithm to estimate precision matrices. In both methods, the convergence thresholds for the EM algorithm and the ADMM algorithm are set to 0.01. As shown in Figure 1, AJGM exhibits a significant computational speed advantage over the comparison methods at different p-values (i.e., gene numbers), with the advantage becoming more pronounced as the number of genes increases.

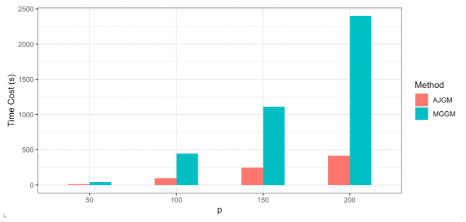

Figure 1: The running time of the two methods on datasets with different gene numbers.

S9. Simulation study on the impact of precision matrix order on the accuracy of network estimation.

In this section, we first discuss whether adjacent dissimilar networks influence the estimation. Following the data generation method described in Section S4 of the Supplementary Material, we generated data for $k = 3$ and $n_k = 300$. The precision matrices were generated manually, with the network structure of subtype 1 and subtype 3 exhibiting higher similarity to each other, while having lower similarity with subtype 2. The precision matrices for the three subtypes were generated as follows: $\Omega_1 = (3M_s, 3M_c, 3M_d, M_0)\Omega_2 = (3M_d, 3M_s, 3M_c, M_d)\Omega_3 = (3M_s, 3M_c, 3M_d, M_c)$

The estimation results are shown in the heatmap in Figure 1, where all three subtypes accurately identify the edges of each module. The relationship identification of subtype 2 is not affected by interference from adjacent networks. Meanwhile, subtype 1 and subtype 3 maintained a very high level of similarity.

We then discuss the impact of the ordering of the precision matrices on network estimation. Simulated data are generated with four subtypes: Networks 1 and 2 share six blocks, Networks 3 and 4 share six blocks, and all four networks share an additional two blocks. This process is repeated 10 times to generate multiple simulated datasets. We test different arrangements of precision matrices and compare their network estimation performance. As shown in Table 1, the results indicate minimal differences in network estimation accuracy across

the three arrangements, with nearly no variation in the estimation of shared relationships.

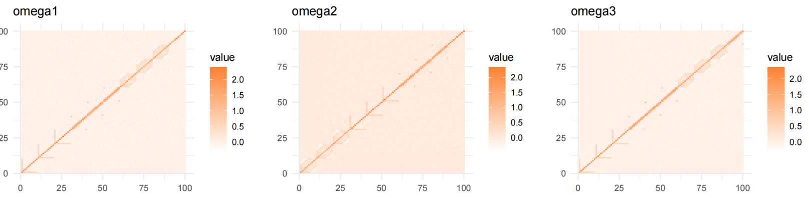

Figure 1: Heatmap of the estimated precision matrices.

Table 1: Network estimation accuracy under different precision matrix orderings.

ordering	edge type	FPR	TPR	F1
1-2-3-4	shared edges	0.00 (0.00)	0.87 (0.04)	0.84 (0.02)
	all edges	0.00(0.00)	0.85 (0.05)	0.82 (0.01)
1-4-2-3	shared edges	0.00(0.00)	0.90 (0.03)	0.87 (0.02)
	all edges	0.01(0.00)	0.86 (0.03)	0.81 (0.03)
1-3-4-2	shared edges	0.00(0.00)	0.88 (0.04)	0.86 (0.03)
	all edges	0.00(0.00)	0.82 (0.03)	0.80 (0.03)

S10. List of 79 genes used in real application.

ANLN	BRCA1	CDH1	FOXC1
CDH3	MAP2K4	CBFB	UBE2T
PGR	RAD54L	BIRC5	ESR1
PHGDH	CDC6	MAP3K1	JAK2
MMP11	MYBL2	TUBB1	NBN
SFRP1	CCNE1	AKT2	BLVRA
BAG1	GATA3	CDKN1B	CCND3
SF3B1	MLPH	CDC20	CENPF
KRT17	FOXA1	ACTR3B	CCNB1
PTPN22	TBX3	MDM2	BRIP1
MYC	CEP55	BRCA2	RB1
SLC39A6	TP53	ERBB2	GRB7
AKT1	KIF2C	PIK3R1	EGFR
MKI67	ATM	CHEK1	TMEM45B
PTPRD	AFF2	FGFR4	PTTG1
MELK	MAP2K1	BUB1	NAT1
CXXC5	BCL2	RRM2	PTEN
GPR160	EXO1	UBE2C	TYMS
AURKB	CHEK2	KRT5	KRT14
MAPT	NF1	MIA	

S11. Downstream biological analysis for TNBC in Section Real Applications.

To address the concerns raised regarding the neglect of heterogeneity and the lack of integration of the overall context in gene network construction, we present an empirical analysis comparing our model, AJGM, with two widely used gene network construction methods: JGL[10] and WGCNA[11]. Among these methods, WGCNA constructs gene networks for each subtype independently, while JGL jointly constructs gene networks for heterogeneous samples with predefined clustering results. In contrast, AJGM simultaneously performs subtype identification and network construction, focusing on shared relationships during network construction and adaptively adjusting the similarity between networks. The subtype classification results for WGCNA and JGL are based on the output of AJGM.

We first investigate the networks estimated by the three methods. Since WGCNA estimates gene networks for each subtype independently, the resulting networks contain more edges specific to each subtype, as shown in Figure 1. In contrast, the jointly estimated gene networks in JGL and AJGM include more shared edges. Additionally, because the incorporation of the overall network in AJGM estimation, its network estimation contains the most edges shared across all subtypes.

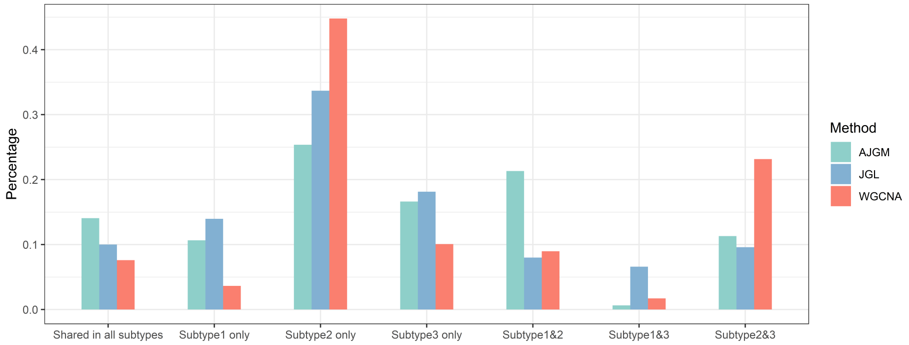

Figure 1: Proportions of different types of relationships in gene networks constructed from scRNA-seq data of triple-negative breast cancer using AJGM and two comparative methods.

As the results of gene network estimation significantly impact downstream biological significance analysis, we perform enrichment analysis on the hub genes identified in the networks estimated by the three methods. For the gene networks constructed using the three methods, genes with a degree greater than 4 in the network are selected as hub genes, and enrichment analysis is performed on them. First, we investigate the network of common relationships for each method, which reflects shared features among different subtypes of TNBC. In the enrichment analysis results of AJGM, several pathways related to the acti-

vation of the cell cycle and processes promoting cell proliferation and division are identified, including *Cell Cycle Checkpoint Signaling*, *Positive Regulation of Cell Cycle Process*, *Mitotic Cell Cycle Checkpoint Signaling*, and *Regulation of Mitotic Metaphase/Anaphase Transition*. These pathways are crucial for the uncontrolled proliferation and aggressive behavior of TNBC. Moreover, the analysis also highlights *Epithelial to Mesenchymal Transition*, a key process associated with the metastatic potential of TNBC, indicating its role in driving tumor invasion and spread[12].

In contrast, networks constructed by JGL and WGCNA reveal pathways associated with the negative regulation of the cell cycle and proliferation, such as *Negative Regulation of Cell Cycle*, *Negative Regulation of Cell Cycle Process*, and *Negative Regulation of Mitotic G2/M Transition*. These pathways, however, are less directly relevant to the characteristic aggressive traits of TNBC. The findings from JGL and AJGM also show a higher degree of similarity in their network estimates and enrichment analysis results, as both methods use joint estimation of Gaussian graphical models. Notably, some pathways, such as *Mitotic Cell Cycle Phase Transition*, appear in the enrichment analysis of both methods. These pathways are often dysregulated in cancer cells, contributing to aberrant cell proliferation and genomic instability, which are hallmark features of tumor progression.

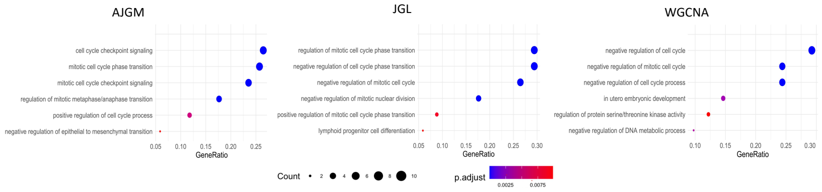

Figure 2: The enrichment analysis results of the network of shared relationships.

In addition, we perform enrichment analysis on the network for subtype-specific relationships. Specifically, we focus on the network corresponding to Subtype 2, as it contains significantly more edges than other subtypes, potentially representing a more aggressive subtype with higher biological significance. Compared to other methods, the enrichment analysis results from AJGM include more biologically meaningful pathways, such as regulation of apoptosis, drug resistance, and pathways commonly observed in TNBC, such as signal transduction by p53 class mediator resulting in cell cycle arrest. These findings highlight that prioritizing shared relationships in the model enhances the understanding of heterogeneity, as shared edges can be directly disregarded when studying heterogeneity.

S12. Real application on application on Arabidopsis root sequencing data.

The root of Arabidopsis thaliana has become a powerful model system for studying gene networks due to its simple anatomy and well-defined developmental trajectories. Root cells are organized longitudinally along a developmental axis, originating from the stem cell niche at the root tip. As cells divide and differentiate, they are displaced shootward, forming a continuum of developmental stages that allows for detailed tracking of gene expression dynamics over time[13]. In this study, we focus on the procambium, a critical stem cell population in the Arabidopsis root responsible for producing vascular tissues. By leveraging scRNA-seq data across multiple developmental timepoints, we construct gene regulatory networks to elucidate the dynamic molecular mechanisms underlying procambium development.

The data are derived from the study by Rachel Shahan et al. [14]. In this study, cells labeled as procambium type in the dataset are selected, totaling 12,905 samples. Based on previous studies and research related to the growth and development of Arabidopsis root, 80 genes closely associated with the biological functions of procambium-type cells, such as development, differentiation, and signal transduction, are chosen for further analysis. PBIC calculation results indicate the presence of three subtypes within the data, which can be assigned as early, intermediate, and late cells based on their developmental time points.

To apply AJGM to temporal data, modifications to the model are required. Since cells in adjacent time points exhibit higher similarity, it is necessary to ensure that the precision matrices in the penalty term are arranged sequentially, from early to intermediate and then to late. For instance, the network of early cells should exhibit higher similarity to that of intermediate cells than to late cells. Fortunately, the sample already contains prior information about the cell's time point. Specifically, each cell in the dataset is labeled with developmental time points ranging from early to late, represented as T0–T9, spanning a total of 10 time stages. These labels can be utilized during parameter inference to determine the time stage of each subtype and dynamically adjust the ordering of the precision matrices. During parameter inference, the three subtypes are sorted based on the mean of the prior period in their corresponding samples to determine whether they belong to the early, middle, or late stage.

Regarding the clustering outcomes, the subtyping results from AJGM align closely with the pre-existing labels in the dataset, demonstrating a strong consistency. Notably, cells from different stages within each of the three subtypes exhibit marked differences, underscoring the robustness of AJGM in accurately classifying cell stages.

In the context of network inference, the hub genes identified in the early-stage network are frequently linked to fundamental processes such as cell development, division, and the initial stages of differentiation. One prominent hub gene, CLE3, plays a pivotal role in regulating early cell development by functioning as a signaling molecule that maintains stem cell populations [15].

This gene is crucial for coordinating cell differentiation and proliferation, which are essential for the proper formation of tissues at the early stages. Similarly, the hub gene WUSCHEL is integral to the regulation of stem cell maintenance and differentiation within meristems. Specifically, it controls early root development by influencing stem cell activity in the root meristem, with a particular focus on the quiescent center [16]. Another key hub gene, CYCD3, is critical in regulating cell proliferation and the transition into differentiation, processes that are fundamental to the growth of early root cells and the development of the root meristem [17].

As the procambium progresses through the mid and late-stages of differentiation into xylem and phloem, more intricate gene pathways are activated, driving cell differentiation, tissue formation, and cellular specialization. For instance, RHD2 is essential for root hair cell differentiation in Arabidopsis, particularly in the elongation and differentiation zones. It regulates the production of reactive oxygen species and calcium influx, both of which are crucial for root hair development [18]. Additionally, HDG12 plays a significant role in the later stages of root development in Arabidopsis, modulating the expression of cell-wall-loosening proteins and ensuring the coordination of cell-wall extensibility with overall root growth[19]. Another hub gene, SHA, regulates signaling pathways in late-stage root cell differentiation, supporting cellular development and helping the plant respond to environmental stresses.

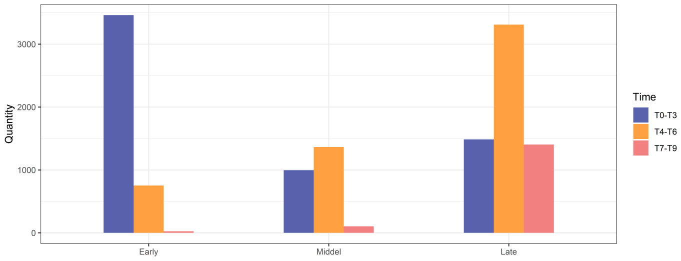

Figure 1: The prior time label distribution corresponding to early, middle, and late-stage cells.

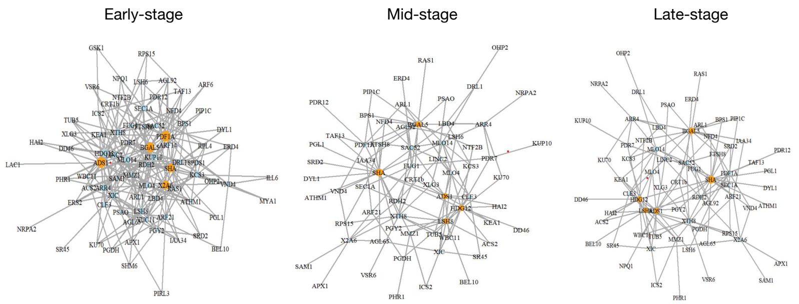

Figure 2: Gene networks of cells at three stages.

S13. Real application on sequencing data of mouse embryonic stem cells.

In this study, we use single-cell RNA sequencing data of mouse embryonic stem cells from Klein et al[20] to investigate cell differentiation processes. The dataset, available in the GEO database (ID: GSE65525), focuses on leukemia inhibitory factor (LIF) as the experimental variable. LIF is known to regulate stem cell pluripotency and inhibit differentiation by modulating processes such as cell division and differentiation. The original data were collected at four time points: before injection (day 0) and 2, 4, and 7 days after injection. For this analysis, we selected samples from day 0 (stage 1) and day 7 (stage 2) to explore the hub genes that influence stem cell growth rates at these two stages. As for gene selection, we focus on 81 genes stem cell markers[21] for the research. The PBIC calculation results indicate that there are four subtypes in both stage 1 and stage 2.

For sequencing data with heterogeneity, we focus on the changes in the network for shared relationships, as this network reflects the changes in the regulatory relationships of the overall cells before and after injection. Compared to Day 0, the gene network at Day 7 exhibits a significant reduction in the degree of genes associated with cell differentiation, as LIF regulates stem cell pluripotency and suppresses differentiation.

At Day 7, LEFTY2 experiences a decline in its degree (Degree Difference = -8), suggesting a weakening of its regulatory influence. LEFTY2 is a critical factor in maintaining the undifferentiated state of stem cells. The reduction in its expression in response to LIF may be linked to the initiation of stem cell differentiation into specific lineages, as LIF encourages differentiation. Similarly, CDX2 shows a decrease in its degree (Degree Difference = -4). This could imply a reduced involvement in differentiation. As a transcription factor associated with embryonic differentiation, the decline in CDX2 regulation in response to LIF underscores LIF's role in keeping stem cells in an undifferentiated state, preventing excessive differentiation. COL1A1 also shows a reduction in its degree (Degree Difference = -5), pointing to a weaker regulatory association. As a key gene in collagen production, COL1A1 plays an essential role in extracellular matrix formation. The decrease in its regulatory connection in response to LIF suggests that LIF inhibits cell differentiation and promotes self-renewal by downregulating COL1A1 expression. Further details about the real-world application can be found in Section S13 of the Supplementary Material.

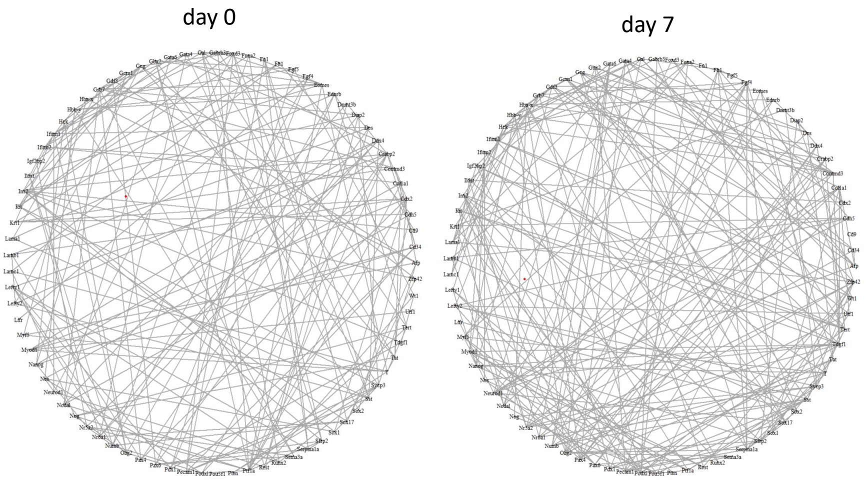

Figure 1: Gene networks of shared relationship in mouse stem cells on day0 and day7.

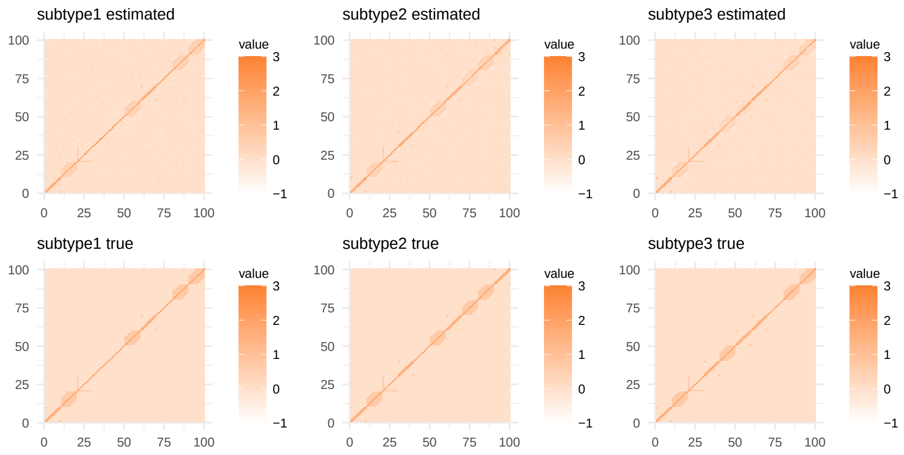

Figure S1: The estimated precision matrices by AJGM and the true precision matrices in simulation study when $p = 100$ and $K = 3$.

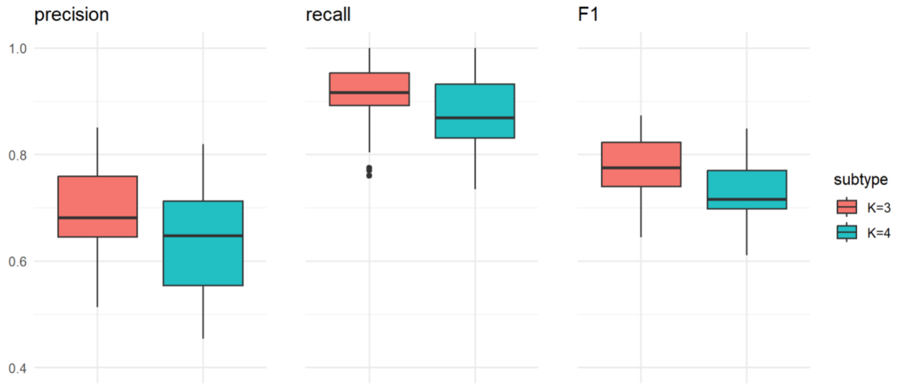

Figure S2: Precision, recall and F1 of the estimated overall network in simulation study.

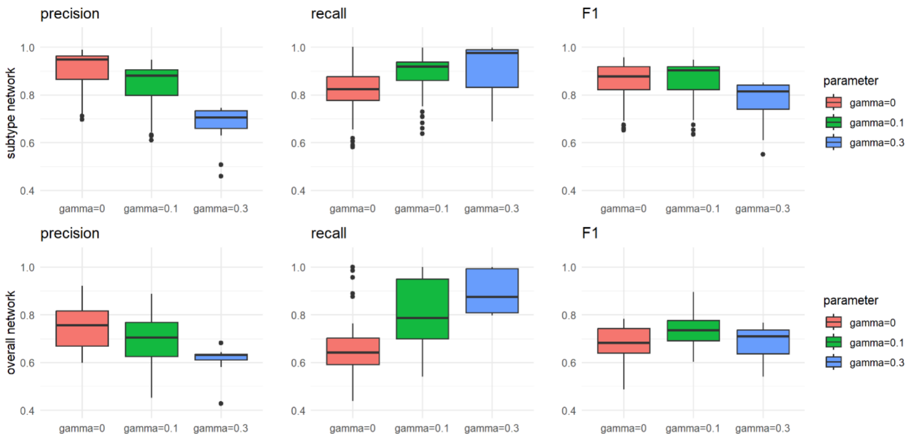

Figure S3: Keeping other parameters constant , the network's inference precision, recall, and F1 scores vary with different values of γ in simulation study.

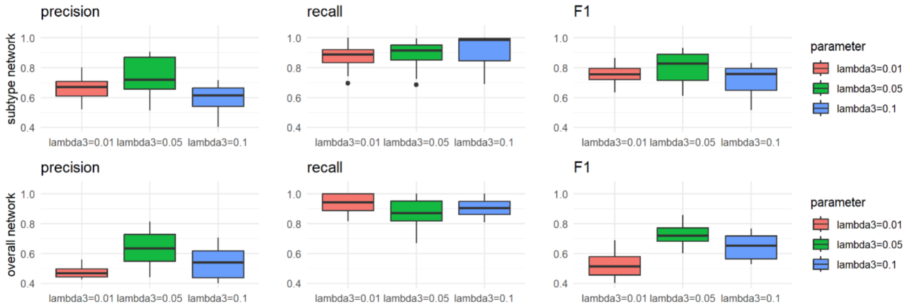

Figure S4: Keeping other parameters constant ($\lambda_2 = 0.05$), the network's inference precision, recall, and F1 scores vary with different values of λ_3 in simulation study.

In Figure S3, $\gamma = 0$ indicates that adaptive weights $w_{ij}^{(k,k+1)}$ are not used in joint estimation. The results demonstrate that appropriate values of γ in adaptive weights can enhance the network estimation performance.

Figure S4 demonstrate that appropriate values of λ_3 in adaptive weights $f(\theta_{ij}^{(1)}, ..., \theta_{ij}^{(k)})$ can enhance the network estimation performance, particular for the overall network.

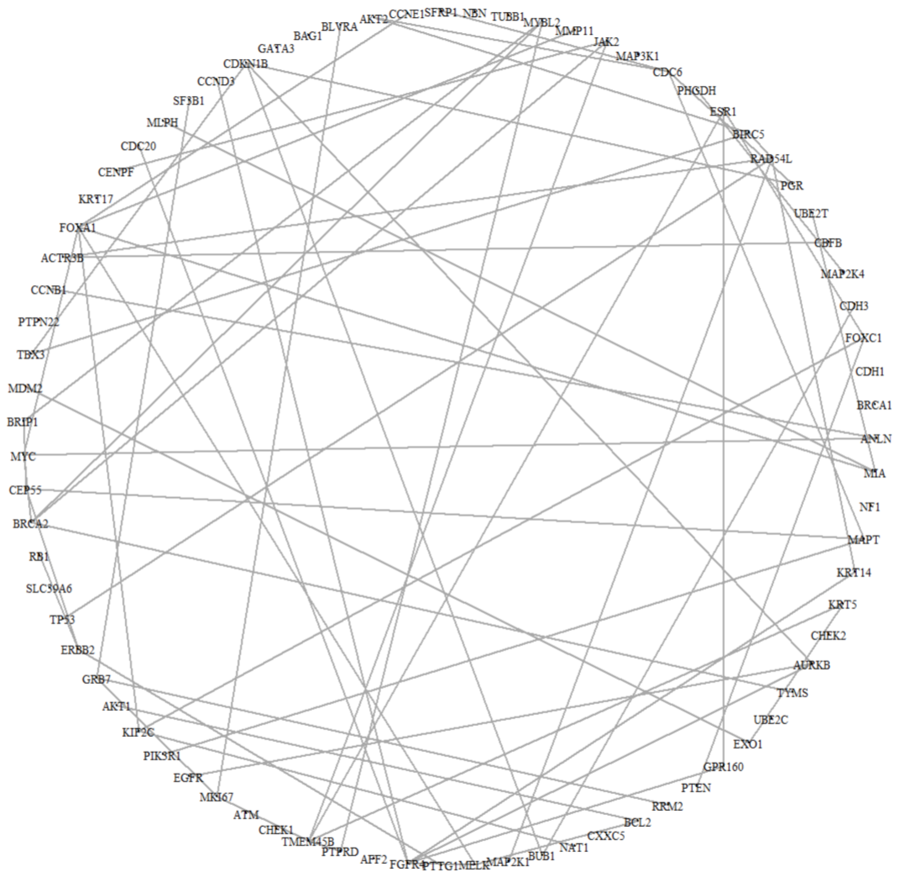

Figure S5: The gene network of shared relationships in triple-negative breast cancer constructed using scRNA-seq data.

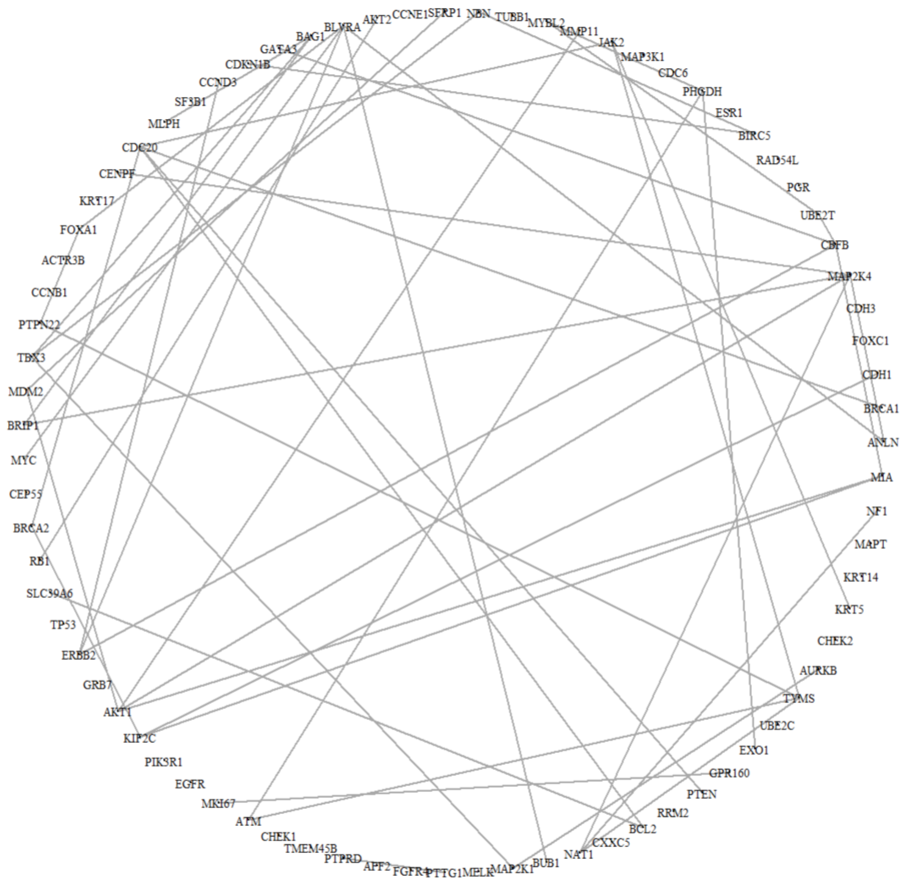

Figure S6: The subtype 1 specific network of triple-negative breast cancer constructed using scRNA-seq data.

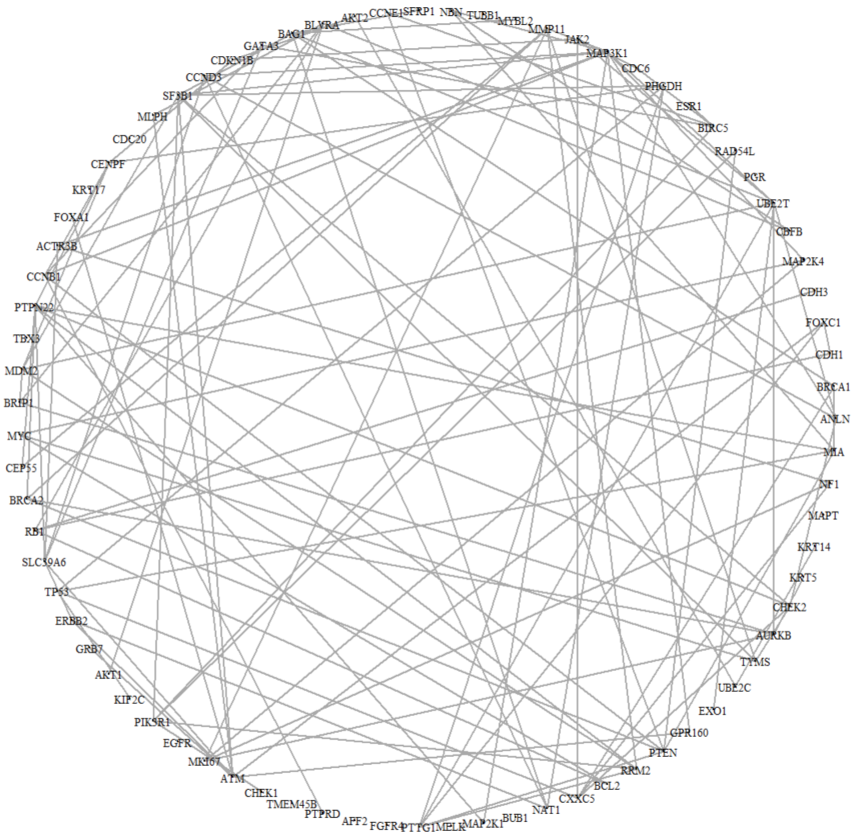

Figure S7: The subtype 2 specific network of triple-negative breast cancer constructed using scRNA-seq data.

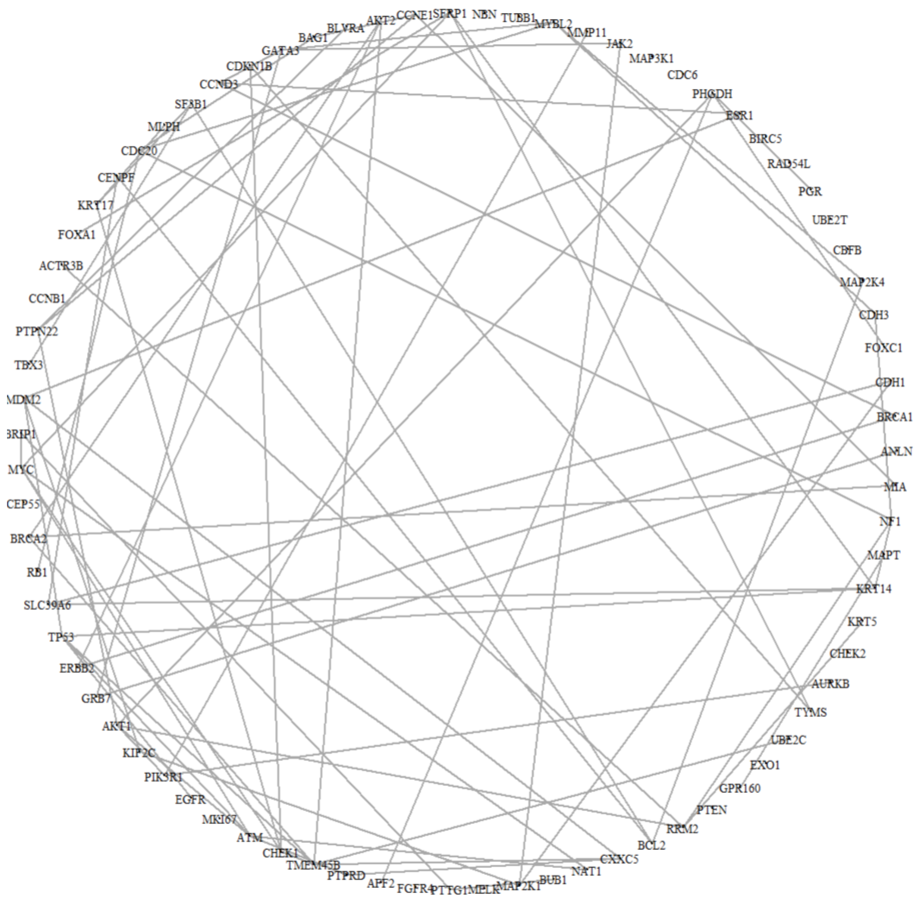

Figure S8: The subtype 3 specific network of triple-negative breast cancer constructed using scRNA-seq data.

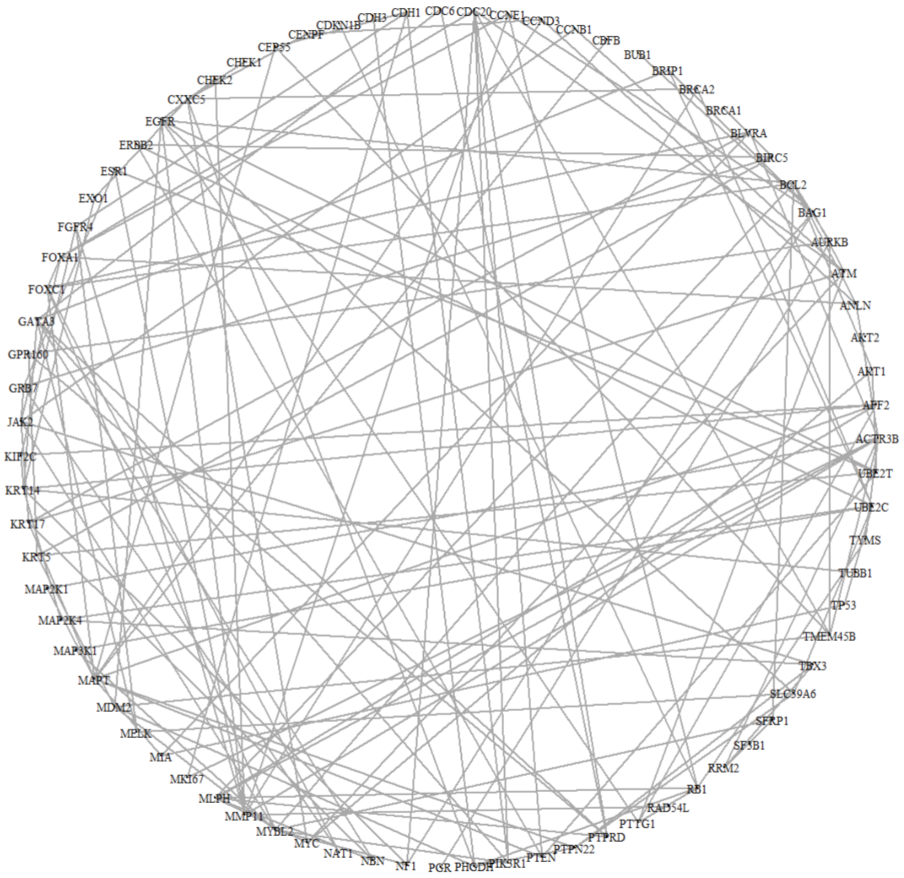

Figure S9: The network of shared relationships in triple-negative breast cancer constructed using Bulk RNA-seq data.

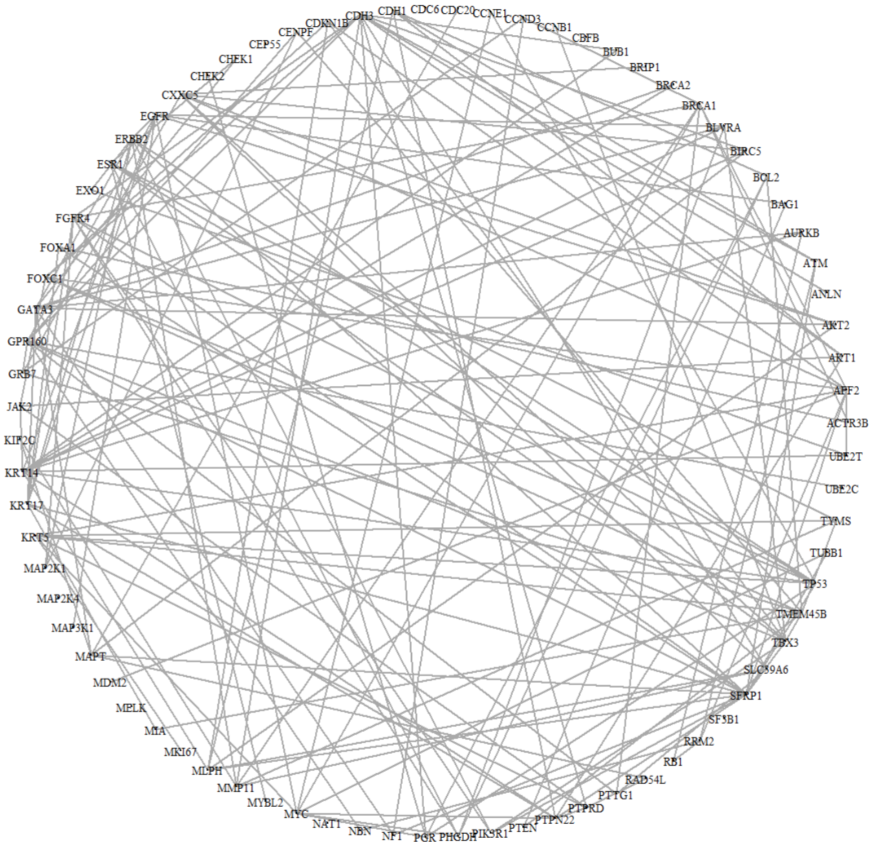

Figure S10: The subtype 1 specific network of triple-negative breast cancer constructed using Bulk RNA-seq data.

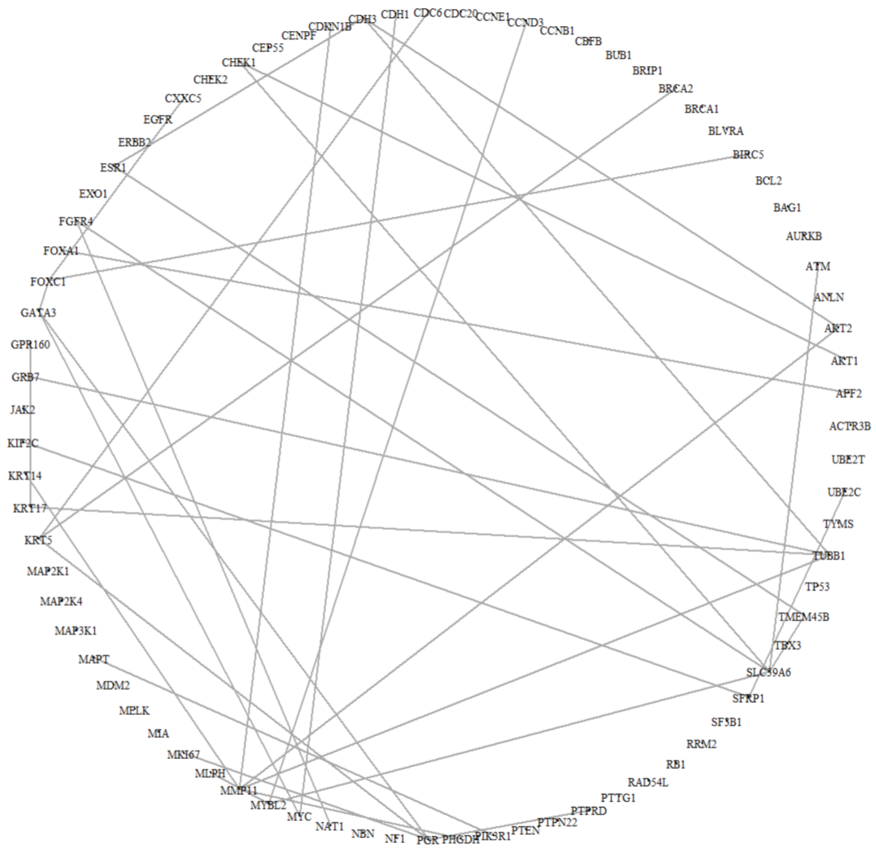

Figure S11: The subtype 2 specific network of triple-negative breast cancer constructed using Bulk RNA-seq data.

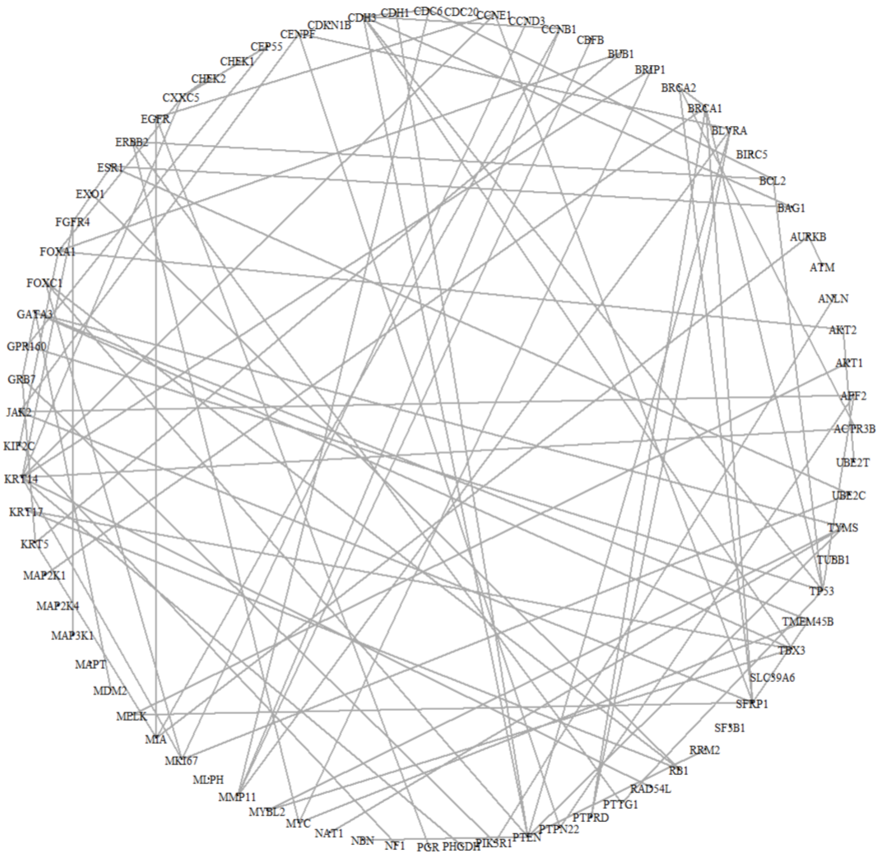

Figure S12: The subtype 3 specific network of triple-negative breast cancer constructed using Bulk RNA-seq data.

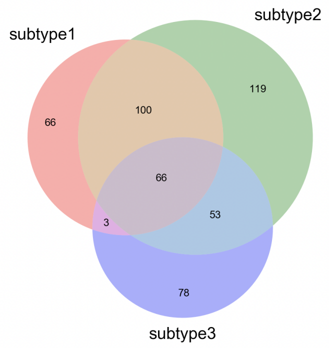

Figure S13: Number of shared edges and specific edges among different subtypes of gene networks constructed using single-cell RNA-seq data.

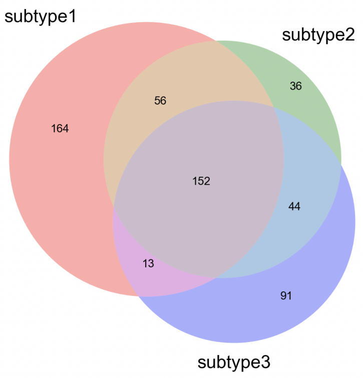

Figure S14: Number of shared edges and specific edges among different subtypes of gene networks constructed using Bulk RNA-seq data.

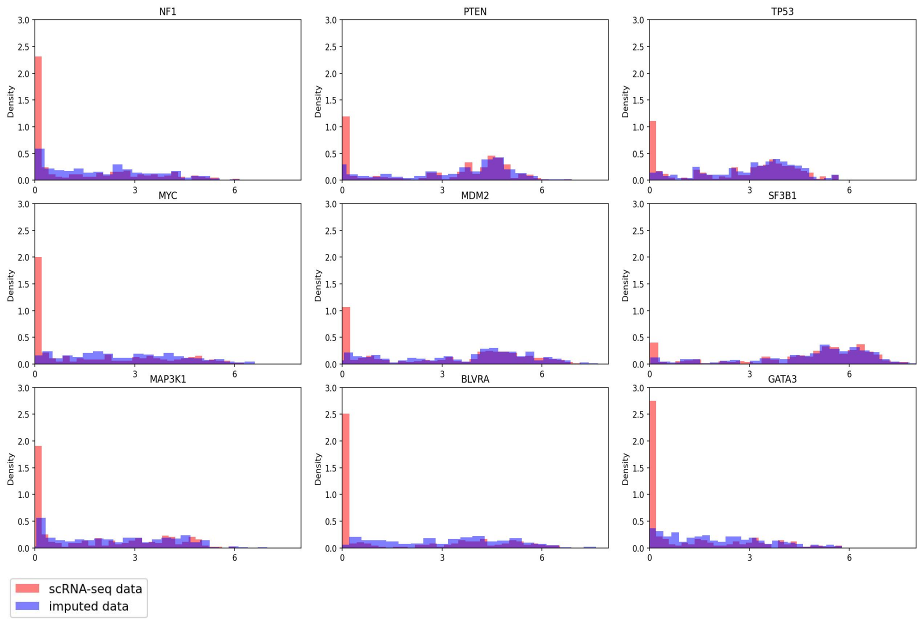

Figure S15: The histograms of scRNA-seq and imputed data for 9 genes in the real application of TNBC.

As shown in the figure, scRNA-seq data with zero inflation approximate a normal distribution after sampling, which satisfies the model assumption of Gaussian graphical models (GGM).

Table S1(a). Performance metrics of zero-inflated data generated by the first method in the simulation study.

	α	AJGM	BLGGM	SCGGM	SILGGM
FPR	0.5	0.02(0.00)	0.01(0.00)	0.01(0.00)	0.02(0.00)
	1	0.01(0.00)	0.01(0.00)	0.01(0.00)	0.01(0.00)
	1.5	0.01(0.00)	0.01(0.00)	0.01(0.00)	0.02(0.00)
TPR	0.5	0.50(0.06)	0.31(0.07)	0.32(0.05)	0.24(0.02)
	1	0.78(0.07)	0.52(0.16)	0.56(0.03)	0.54(0.03)
	1.5	0.81(0.04)	0.54(0.10)	0.62(0.05)	0.76(0.05)
F1	0.5	0.57(0.05)	0.46(0.08)	0.48(0.04)	0.38(0.02)
	1	0.79(0.04)	0.60(0.11)	0.70(0.03)	0.65(0.04)
	1.5	0.82(0.04)	0.64(0.09)	0.74(0.03)	0.73(0.02)

Table S1(b). Performance metrics of zero-inflated data generated by the second method in the simulation study.

	β	AJGM	BLGGM	SCGGM	SILGGM
FPR	0.4	0.00 (0.00)	0.01 (0.00)	0.00 (0.00)	0.00 (0.00)
	0.6	0.00 (0.00)	0.00 (0.00)	0.01 (0.00)	0.01 (0.00)
	0.8	0.00 (0.00)	0.00 (0.00)	0.00 (0.00)	0.00 (0.00)
TPR	0.4	0.78 (0.06)	0.63 (0.04)	0.59 (0.05)	0.64 (0.04)
	0.6	0.79 (0.06)	0.68 (0.05)	0.67 (0.02)	0.74 (0.03)
	0.8	0.83 (0.04)	0.69 (0.02)	0.71 (0.03)	0.80 (0.01)
F1	0.4	0.74 (0.07)	0.77 (0.03)	0.66 (0.06)	0.69 (0.02)
	0.6	0.80 (0.03)	0.75 (0.02)	0.70 (0.03)	0.75 (0.03)
	0.8	0.84 (0.03)	0.78 (0.03)	0.76 (0.04)	0.82 (0.03)

Table S2. Performance metrics of models for two overall sample generation methods.

	100 (Sample Size)	300 (Sample Size)	900 (Sample Size)	Set $X^{'}$ as X
FPR	0.01(0.01)	**0.01(0.00)**	0.02(0.00)	**0.01(0.00)**
TPR	0.76(0.05)	0.87(0.08)	0.84(0.05)	**0.90(0.04)**
F1	0.76(0.05)	0.85(0.08)	0.74(0.03)	**0.89(0.03)**

As shown in Table S2, the subtype network estimation performance of obtaining $x_l^{'}$ from sampling varies across different sample size, all of which are lower than that of setting $x_l^{'}$ as x_l.

Table S3. Performance metrics of obtaining $x_l^{'}$ from sampling with zero-inflated data in simulation study (sample size is 300).

	α	AJGM
	0.5	0.00(0.00)
FPR	1	0.00(0.00)
	1.5	0.01(0.00)
	0.5	0.43(0.09)
TPR	1	0.72(0.04)
	1.5	0.76(0.05)
	0.5	0.55(0.06)
F1	1	0.82(0.03)
	1.5	0.83(0.03)

Table S4. Performance metrics of public and nonpublic blocks.

	block type	AJGM	BLGGM	GGMPF	MGGM	SCGGM	SILGGM
FPR	public	0.01(0.00)	0.00(0.00)	0.02(0.00)	0.04(0.00)	0.01(0.00)	0.01(0.00)
	nonpublic	0.01(0.00)	0.01(0.00)	0.02(0.00)	0.03(0.00)	0.01(0.00)	0.01(0.00)
TPR	public	0.99(0.01)	0.64(0.08)	0.77(0.06)	0.77(0.12)	0.775(0.04)	0.832(0.06)
	nonpublic	0.91(0.08)	0.71(0.06)	0.81(0.08)	0.82(0.08)	0.656(0.03)	0.854(0.04)
F1	public	0.95(0.01)	0.81(0.06)	0.86(0.04)	0.76(0.07)	0.823(0.03)	0.783(0.04)
	nonpublic	0.87(0.07)	0.76(0.10)	0.88(0.09)	0.79(0.05)	0.755(0.02)	0.817(0.03)

Table S5. The hub genes in subtype shared network of triple-negative breast cancer constructed using scRNA-seq data.

Hub genes	Gene degree
FOXA1	7
FGFR4	7
RAD54L	6
TMEM45B	6
CDC6	5
CDKN1B	5
BRCA2	5
FOXC1	4

Table S6. The hub genes in the triple-negative breast cancer subtype specific networks constructed using scRNA-seq data.

Hub genes of subtype1	Gene degree	Hub genes of subtype2	Gene degree	Hub genes of subtype3	Gene degree
MAP2K4	5	MAP3K1	11	TMEM45B	7
BLVRA	5	UBE2T	8	MYC	6
CDC20	5	SF3B1	8	TP53	6
CBFB	4	PTPN22	8	AKT1	6
AKT1	4	ATM	8	ATM	6
TYMS	4	MMP11	6	PHGDH	5
PHGDH	3	BAG1	6	MYBL2	5
JAK2	3	SLC39A6	6	SFRP1	5

Table S7. The hub genes in subtype shared network of triple-negative breast cancer constructed using Bulk RNA-seq data.

Hub genes	Gene degree
MAPT	10
ACTR3B	9
MMP11	8
MYBL2	8
FOXA1	7
FOXC1	7
EGFR	7
GATA3	7

Table S8. The hub genes in the triple-negative breast cancer subtype specific network constructed using Bulk RNA-seq data.

Hub genes of subtype1	Gene degree	Hub genes of subtype2	Gene degree	Hub genes of subtype3	Gene degree
SFRP1	15	MMP11	5	KRT14	8
CDH3	12	SLC39A6	5	PTEN	7
AFF2	9	PGR	4	CDH3	6
FGFR4	9	TUBB1	4	SFRP1	6
TMEM45B	9	CDH3	3	TP53	6
EGFR	9	FOXC1	3	BRCA1	5
ERBB2	9	GATA3	3	GATA3	5
GATA3	9	KRT5	3	BLVRA	4

References

[1] Souvik Seal, Qunhua Li, Elle Butler Basner, Laura M Saba, and Katerina Kechris. Rcfgl: Rapid condition adaptive fused graphical lasso and application to modeling brain region co-expression networks. *PLoS computational biology*, 19(1):e1010758, 2023.

[2] Daniela M Witten and Robert Tibshirani. Covariance-regularized regression and classification for high dimensional problems. *Journal of the Royal Statistical Society Series B: Statistical Methodology*, 71(3):615–636, 2009.

[3] Robert Tibshirani, Michael Saunders, Saharon Rosset, Ji Zhu, and Keith Knight. Sparsity and smoothness via the fused lasso. *Journal of the Royal Statistical Society Series B: Statistical Methodology*, 67(1):91–108, 2005.

[4] Qiuyu Wu and Xiangyu Luo. Estimating heterogeneous gene regulatory networks from zero-inflated single-cell expression data. *The Annals of Applied Statistics*, 16(4):2183–2200, 2022.

[5] Tan. *Research on gene network inference method and application based on probabilistic graph modeling.* PhD thesis, Central China Normal University, 2022.

[6] Mingyang Ren, Sanguo Zhang, Qingzhao Zhang, and Shuangge Ma. Gaussian graphical model-based heterogeneity analysis via penalized fusion. *Biometrics*, 78(2):524–535, 2022.

[7] Chen Gao, Yunzhang Zhu, Xiaotong Shen, and Wei Pan. Estimation of multiple networks in gaussian mixture models. *Electronic journal of statistics*, 10:1133, 2016.

[8] Wei Vivian Li and Jingyi Jessica Li. An accurate and robust imputation method scimpute for single-cell rna-seq data. *Nature communications*, 9(1):997, 2018.

[9] Rong Zhang, Zhao Ren, and Wei Chen. Silggm: An extensive r package for efficient statistical inference in large-scale gene networks. *PLoS computational biology*, 14(8):e1006369, 2018.

[10] Patrick Danaher, Pei Wang, and Daniela M Witten. The joint graphical lasso for inverse covariance estimation across multiple classes. *Journal of the Royal Statistical Society Series B: Statistical Methodology*, 76(2):373–397, 2014.

[11] Peter Langfelder and Steve Horvath. Wgcna: an r package for weighted correlation network analysis. *BMC bioinformatics*, 9:1–13, 2008.

[12] Sai Yin Cheung, Yvonne Jia Yu Boey, Valerie Cui Yun Koh, Aye Aye Thike, Jeffrey Chun Tatt Lim, Jabed Iqbal, and Puay Hoon Tan. Role of epithelial–mesenchymal transition markers in triple-negative breast cancer. *Breast cancer research and treatment*, 152:489–498, 2015.

[13] Liam Dolan, Kees Janmaat, Viola Willemsen, Paul Linstead, and Ben Scheres. Cellular organisation of the arabidopsis thaliana root. *Development*, 119(1):71–84, 1993.

[14] Rachel Shahan, Che Wei Hsu, Trevor M. Nolan, Benjamin J. Cole, Isaiah W. Taylor, Laura Greenstreet, Stephen Zhang, Anton Afanassiev, Anna Hendrika Cornelia Vlot, and Geoffrey Schiebinger. A single-cell arabidopsis root atlas reveals developmental trajectories in wild-type and cell identity mutants. *Developmental Cell*, 57(4):543–560.e9, 2022.

[15] Guodong Wang, Guohua Zhang, and Mengyao Wu. Cle peptide signaling and crosstalk with phytohormones and environmental stimuli. *Frontiers in plant science*, 6:1211, 2016.

[16] Yuguang Song, Xinru Ding, Xueying Sun, Zhaoran Zhang, and Wei Dong. Aba inhibits in vitro shoot regeneration by affecting h3k9ac modification ofwusinarabidopsis. *Protoplasma*, 261(6):1327–1333, 2024.

[17] Baekelandt Alexandra, Pauwels Laurens, Wang Zhibiao, Li Na, De Milde Liesbeth, Natran Annelore, Vermeersch Mattias, Li Yunhai, Goossens Alain, and Inzé Dirk. Arabidopsis leaf flatness is regulated by ppd2 and ninja through repression of cyclin d3 genes. *Plant physiology*, 178:pp.00327.2018, 2018.

[18] Tomáš Takáč, Lenka Kuběnová, Olga Šamajová, Petr Dvořák, Jan Řehák, Jan Haberland, Sebastian T. Bundschuh, Tibor Pechan, Pavel Tomančák, Miroslav Ovečka, and Jozef Šamaj. Actin cytoskeleton and plasma membrane aquaporins are involved in different drought response of arabidopsis rhd2 and der1 root hair mutants. *Plant Physiology and Biochemistry*, 216:109137, 2024.

[19] Sayuri Tanaka, Yuuki Matsushita, Yuga Hanaki, Takumi Higaki, Naoya Kamamoto, Katsuyoshi Matsushita, Tetsuya Higashiyama, Koichi Fujimoto, and Minako Ueda. Hd-zip iv genes are essential for embryo initial cell polarization in the radial axis initiation in arabidopsis. *bioRxiv*, pages 2024–04, 2024.

[20] Allon M Klein, Linas Mazutis, Ilke Akartuna, Naren Tallapragada, Adrian Veres, Victor Li, Leonid Peshkin, David A Weitz, and Marc W Kirschner. Droplet barcoding for single-cell transcriptomics applied to embryonic stem cells. *Cell*, 161(5):1187–1201, 2015.

[21] Laralynne M Przybyla and Joel Voldman. Attenuation of extrinsic signaling reveals the importance of matrix remodeling on maintenance of embryonic stem cell self-renewal. *Proceedings of the National Academy of Sciences*, 109(3):835–840, 2012.
